# Occurrence and phenomenology of hallucinations in the general population: A large online survey

**DOI:** 10.1038/s41537-022-00229-9

**Published:** 2022-04-23

**Authors:** Mascha M. J. Linszen, Janna N. de Boer, Maya J. L. Schutte, Marieke J. H. Begemann, Jacqueline de Vries, Sanne Koops, Renske E. Blom, Marc M. Bohlken, Sophie M. Heringa, Jan Dirk Blom, Iris E. C. Sommer

**Affiliations:** 1 Department of Psychiatry & UMC Utrecht Brain Center, University Medical Center Utrecht, Utrecht University, Utrecht, the Netherlands; 2 Section Cognitive Neuroscience, Department of Biomedical Sciences of Cells and Systems, University Medical Center Groningen, University of Groningen, Groningen, the Netherlands; 3 Department of Psychiatry, University Medical Center Groningen, University of Groningen, Groningen, the Netherlands; 4 Parnassia Psychiatric Institute, The Hague, the Netherlands; 5 Reinier van Arkel Mental Health Institute, ‘s Hertogenbosch, the Netherlands; 6 GGZ Centraal, Mental Health Care Institute, Ermelo, the Netherlands; 7 Department of Medical Psychology, Isala Clinics, Zwolle, the Netherlands; 8 Faculty of Social and Behavioural Sciences, Leiden University, Leiden, the Netherlands; 9 Faculty of Biological and Medical Psychology, University of Bergen, Bergen, Norway

**Keywords:** Human behaviour, Psychosis

## Abstract

Although epidemiological studies report that hallucinations occur in 6–15% of the general population, little is known about their phenomenology. To overcome this paucity, this study investigates the phenomenological characteristics of hallucinations in the general population, by using a nationally promoted online survey to assess hallucination phenomenology in four sensory modalities, through a self-report version of the Questionnaire for Psychotic Experiences (QPE), in 10,448 participants (aged 14–88 years). The phenomenology of hallucinations was assessed if hallucinations reportedly occurred in the past month. In the past month, auditory hallucinations were reported most frequently (29.5%), followed by visual (21.5%), tactile (19.9%), and olfactory hallucinations (17.3%); hallucinations in two or more modalities were reported by 47.6%. Substantial numbers of participants rated their hallucinations as severe, due to negative content (16.0–31.6%), previous bothersome experiences (14.8–20.2%), ensuing distress (10.5–16.8%), and/or ensuing disfunctioning (12.7–17.3%). Decreased insight was found in 10.2–11.4%. Hypnagogia was reported by 9.0–10.6%, and bereavement hallucinations by 2.8%. Despite a low prevalence of delusions (7.0%), these phenomena were significantly associated with recent hallucinations, observed in up to 13.4% of the participants with hallucinations during the past week (*p* < 0.001). Our results indicate a wide variety of the phenomenology of hallucinations in the general population and support the existence of a phenomenological continuum.

## Introduction

Although hallucinations are often studied in the context of neurological, psychiatric, or somatic disorders^1^, it is increasingly known as a phenomenon that also occurs in the general population, with prevalence rates of 6–15%^2,3^. Several models of sensory perception in the general population, state that misperceptions, such as hallucinations, illusions, and distortions (Box 1^4,5^,) can be considered a common byproduct of physiological processes that enable rapid sensory perception^6–10^. Studying hallucinations in non-clinical populations increases our understanding of hallucinations and their underlying mechanisms, as it largely bypasses confounding factors that typically occur in clinical populations, such as medication effects and comorbidity.

Examining the phenomenology of hallucinations can provide important anchor points for finding underlying mechanisms and indications for further assessment and, in more severe cases, treatment^5,11–13^. Although the prevalence of hallucinations in the general population has been studied extensively by means of large-scale epidemiological surveys^2,3,14^, the overall phenomenological characteristics of hallucinations in this domain remain understudied, since they are mostly limited to either subpopulations^15–17^ (e.g., university students, elderly people) or specific hallucination types^18–21^. The current study aims to reveal the phenomenology of hallucinations in a large sample from the general population aged 14 years and over. Given the previously established association between hallucinations and delusions in the general population^22^, we also assess the presence of delusions to study this association in more detail.

Box 1 The classification of hallucinations and related perceptual phenomena^4,5^ Hallucinations are perceptual phenomena. The overarching term for these phenomena is ‘misperceptions’ or ‘disorders of perception’, which can be further divided into positive and negative disorders of perception. Negative disorders of perception are characterized by a lack or a deficit, as exemplified by blindness, hemianopia, deafness, anaesthesia, anosmia, and hypogeusia. Positive disorders of perception are characterized by the presence of something ‘extra’, i.e., by something that is not based on sensory information. We distinguish three main groups of positive disorders of perception, comprising hallucinations (percepts without a matching stimulus in the outside world), illusions (percepts with a matching stimulus in the outside world, but one that is either misperceived or misinterpreted, such as a moving curtain being mistaken for a cat), and distortions (percepts with a matching stimulus in the outside world, but with discrete alterations of highly specific aspects, such as vertical lines being perceived as slanted (plagiopsia), straight lines being perceived as wavy (dysmorphopsia), and things being perceived substantially smaller than they are (micropsia)). Since the term ‘positive disorders of perception’ may inadvertently evoke associations with disease entities rather than perceptual symptoms, we chose throughout the present study to stick to its synonym, ‘misperceptions’, when referring to hallucinations, illusions, and distortions.

## Results

### Participants

In total, 10,448 valid entries were recorded. Supplementary Figure 2b shows a flowchart of participation rates and exclusions for each part of the survey. Demographic characteristics are listed in Table 1 for the entire sample, and in Table 2 after stratification and comparison for the most recent occurrence of hallucinations. The median reported age within the included sample was 32 years (IQR 23-47); 68.9% of the entire sample was female. The optional questions on delusions were answered by a majority of participants (*n* = 6523; 62.4%; Supplementary Figs. 1ab, 4).

**Table 1:** Demographic characteristics of the entire study sample (*n* = 10,448).

Category	Items	Total group (*n* = 10,448)
Gender	Female; *n* (%)	7195 (68.9)
	Male; *n* (%)	3253 (31.1)
Handedness	Right; *n* (%)	8776 (84.0)
	Left; *n* (%)	1252 (12.0)
	Both; *n* (%)	420 (4.0)
Native speaker	Yes; *n* (%)	9999 (95.7)
Education level	Low; *n* (%)	1159 (11.1)
	Middle; *n* (%)	3690 (35.3)
	High; *n* (%)	5599 (53.6)
Years of education^a^	md (IQR)	15 (14-15)
	m (SD)	14.0 (2.1)
Age	md (IQR)	32 (23-47)
	m (SD)	35.5 (15.2)

^a^Based on the standardized duration of each educational variant.

**Table 2:** Demographic characteristics after categorization and comparison of participants based on the most recent presence of hallucinations.

		Last occurrence of hallucinations?^a^				
Category	Items	Never^a^ (*n* = 2,075)	Ever, more than 1 month ago^a^ (*n* = 3038)	Past month, but not week^a^ (*n* = 1948)	Past week^a^ (*n* = 3387)	Statistics^b^
Gender	Female; *n* (%)	1112 (53.6)	2060 (67.8)	1413 (72.5)	2610 (77.1)	*p* < 0.001, df 3, χ^2^ 346
	Male; *n* (%)	963 (46.4)	978 (32.2)	535 (27.5)	777 (22.9)	
Handedness	Right; *n* (%)	1759 (84.8)	2586 (85.1)	1619 (83.1)	2812 (83.0)	*p* < 0.005, df 6, χ^2^ 18.5
	Left; *n* (%)	249 (12.0)	351 (11.6)	248 (12.7)	404 (11.9)	
	Both; *n* (%)	67 (3.2)	101 (3.3)	81 (4.2)	171 (5.0)	
Native speaker	Yes; *n* (%)	1998 (96.3)	2916 (96.0)	1863 (95.6)	3322 (95.1)	*p* < 0.168, df 3, χ^2^ 5.06
Education level	Low; *n* (%)	154 (7.4)	302 (9.9)	213 (10.9)	490 (14.5)	*p* < 0.001, df 6, χ^2^ 296
	Middle; *n* (%)	548 (26.4)	987 (32.5)	737 (37.8)	1418 (41.9)	
	High; *n* (%)	1373 (66.2)	1749 (57.6)	998 (51.2)	1479 (43.7)	
Years of education^c^	md (IQR)	15 (14–16)	15 (14–16)	15 (14-15)	14 (12–15)	KW: *p* < 0.001, df 3, K 327. JT: *p* < 0.001, z -17.8.
	m (SD)	14.4 (2.0)	14.1 (2.1)	13.9 (2.1)	13.6 (2.2)	
Age	md (IQR)	37 (25–51)	35 (24–49)	29 (22–45)	28 (21–43)	KW: *p* < 0.001, df 3, K 330. JT: *p* < 0.001, z -18.2.
	m (SD)	39.1 (15.9)	37.3 (15.1)	33.9 (15.0)	32.7 (14.4)	

^a^Subgroups categorized based on the reported last occurrence of hallucinations; never, reported having never experienced hallucinations; ever, more than 1 month ago, reported having ever experienced hallucinations, but not in the past month; past month but not week, reported having experienced hallucinations in the past month, but not in the past week; past week, reported having experienced hallucinations in the past week.

^b^Statistics based on two-sided, comparative analyses between four distinct subgroups (never; ever, more than 1 month ago; past month, but not week; past week). Since age and years of education were not normally distributed, Kruskal-Wallis tests were used for subgroup comparisons.

^c^Based on the standardized duration of each educational variant.

*Df* degree(s) of freedom; *Md* median; *IQR* interquartile range; *KW* Kruskall-Wallis test; *JT* Jonckheere-Terpstra test; *M* mean; *SD* standard deviation of the mean.

### Occurrence of hallucinations

**Fig. 1:**
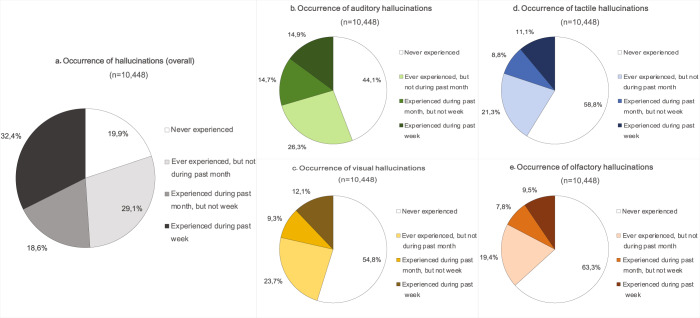
Occurrence of hallucinations. Based on response to screening items from the QPE in the entire study sample (*n* = 10,448), as measured for the auditory (green; **b**), visual (yellow; **c**), tactile (blue; **d**), and olfactory (red; **e**) sensory modalities, as well as an overall category combining the occurrence of hallucinations in all four sensory modalities (gray; **a**).

The survey revealed high percentages of hallucination occurrence (Fig. 1). Auditory hallucinations (AH) were reported most frequently, with 29.4% of the participants (*n* = 3086) having experienced them within the past month, followed by visual hallucinations (VH) (21.5%, *n* = 2248), tactile hallucinations (TH) (19.9%, *n* = 2207) and olfactory hallucinations (OH) (17.3%, *n* = 1807). A more recent experience of hallucinations was significantly associated with female gender, younger age, and a lower education level (Table 2).

### Phenomenology of hallucinations

**Fig. 2:**
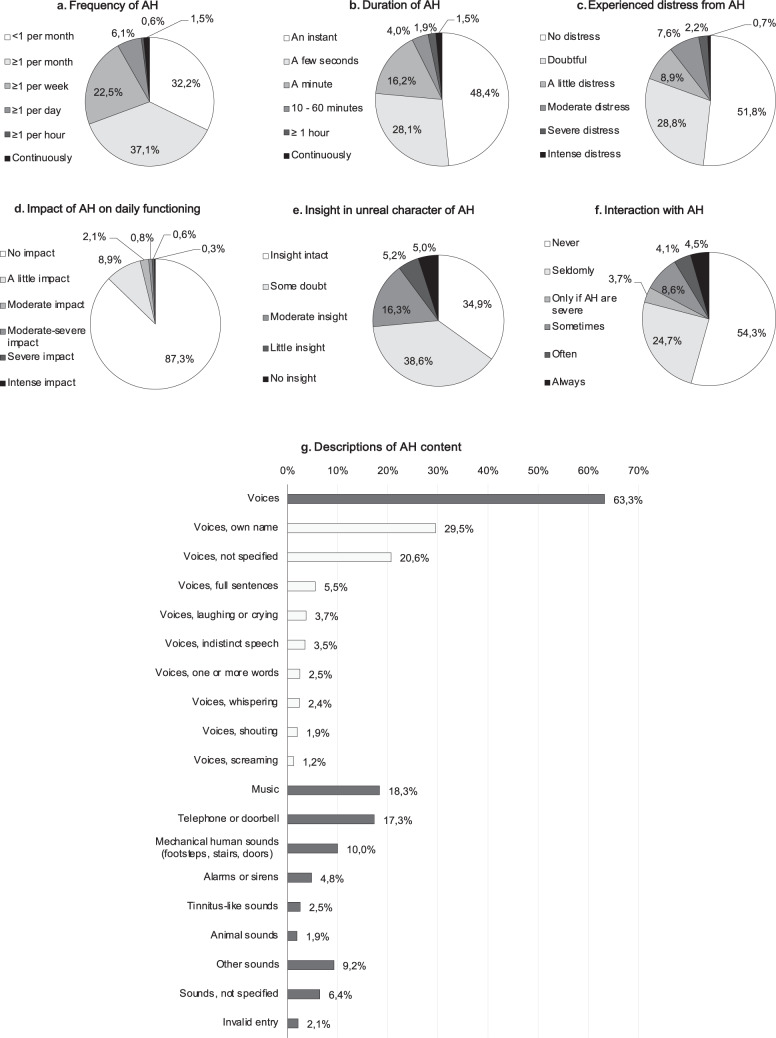
Phenomenology of auditory hallucinations in the past month. Total *n* (AH) = 3086. Additional phenomenological items are listed in Supplementary Fig. 3. Data are listed per QPE item, as obtained through multiple choice items with one answer possibility (**a**–**f**) and an open question, asking participants to describe the experienced content of AH (**g**, total percentage exceeds 100%). Invalid entries AH (**g**): likely external source (*n* = 4); experienced while asleep (*n* = 5); incomplete or incomprehensible information (*n* = 57).

**Fig. 3:**
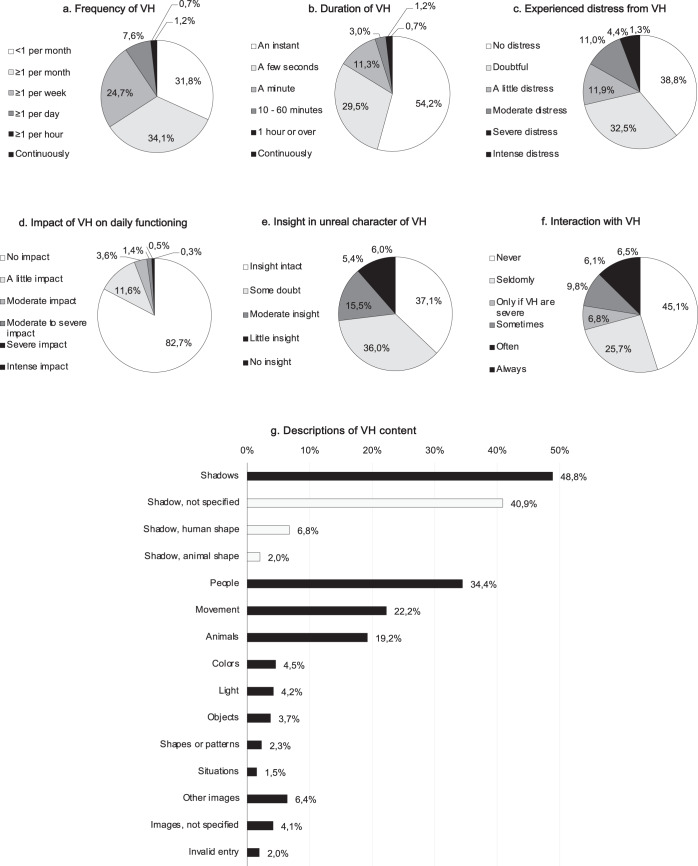
Phenomenology of visual hallucinations in the past month. Total *n* (VH) = 2248. Additional phenomenological items are listed in Supplementary Fig. 4. Data are listed per QPE item, as obtained through multiple choice items withone answer possibility (**a**–**f**) and an open question, asking participants to describe the experienced content of VH (**g**; total percentage exceeds 100%). Invalid entries VH (**g**): incomplete or incomprehensible information (*n* = 44).

**Fig. 4:**
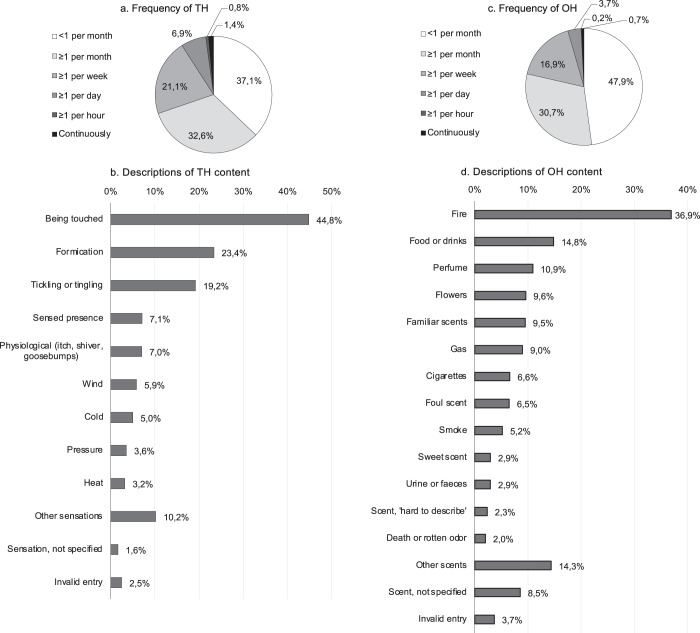
Phenomenology of tactile and olfactory hallucinations in the past month. Phenomenology of tactile hallucinations (**a**, **b**) and olfactory hallucinations (**c**, **d**) in the past month. Total *n* (TH) = 2077; total *n* (OH) = 1807. Additional phenomenological items are listed in Supplementary Fig. 5. Data are listed per QPE item, as obtained through multiple choice items with one answer possibility (**a**, **c**) and open questions, asking participants to describe the experienced content of TH (**b**) or OH (**d**). Some descriptions of TH contained terms that may be difficult to distinguish from regular physiological reactions to internal or external stimuli, such as itch, pain, shivers, or goosebumps, and were therefore categorized as ‘physiological’. Invalid entries TH: likely external source (*n* = 5); incomplete or incomprehensible information (*n* = 47). Invalid entries OH (**d**): likely external source (*n* = 24); incomplete or incomprehensible information (*n* = 42).

Figures 2–4 provide the most relevant items of phenomenology of hallucinations present in the past month, summarized for each sensory modality. Supplementary Figures 3–5 provide additional detailed charts of other phenomenological items. The phenomenology of hallucinations was heterogeneously distributed across QPE-items and modalities. Following the overall structure of the QPE, hallucination phenomenology was most extensively studied in AH and VH.

As for hallucination frequency (Figs. 2a, 3a, 4a, d), the majority of participants experienced hallucinations less than once a week (AH 69.3%; VH 65.1%; TH 69.7%; OH 78.6%), but a smaller, substantial number experienced them at least once daily (AH 8.2%; VH 9.4%; TH 9.1%; OH 4.5%). Likewise, the duration of AH (Fig. 1b) and VH (Fig. 2b) typically lasted only an instant or seconds, but was also described to last for ten or more minutes by a quantity of participants with AH (7.4%) and VH (4.9%). Around one in ten participants indicated that they mostly experienced AH (Fig. 1n) or VH (Fig. 2n) while falling asleep or waking up, indicative of hallucinations of a hypnagogic or hypnopompic nature. For each of the measured sensory modalities, the majority of participants indicated having experienced their hallucinations for 10 years or longer (AH 54.5%; VH 60.3%; TH 54.4%; OH 53.5%) (Supplementary Figs. 3g, 4h, 5c, f).

A small but substantial number of participants indicated that they experienced at least moderate distress as a result of their AH (10.5%; Fig. 2c) or VH (16.8%; Fig. 3c). Similarly, substantial proportions of participants indicated that their AH (12.7%; Fig. 2d) or VH (17.3%; Fig. 3d) at least somewhat impacted their daily functioning.

Regarding insight in the unreal character of the phenomena, 10.2% of participants with AH (Fig. 2e) and 11.4% of those with VH (Fig. 3e) were near to fully convinced that the perceived sounds or images were real. Around half of the hallucinating participants described having at least some interaction with the perceived sounds (45.7%; Fig. 2f) or images (54.9%; Fig. 3f). A subsample described receiving commands from their AH (8.3%; Supplementary Fig. 3c) or VH (5.2%; Supplementary Fig. 4c), on which some participants even acted.

One in every five to six participants with AH, VH, TH, or OH indicated that the content of their experiences was associated with a previous bothersome experience (Supplementary Figs. 3b, 4b, 5a, d). In participants with AH, 34.1% perceived the content of their AH as (partially) negative (Supplementary Fig. 3a); 53.2% of participants with VH experienced the content of the perceived images to be at least somewhat negative (Supplementary Fig. 4a).

For each sensory modality, participants were asked to provide an example of the content of their hallucinations (Figs. 2g, 3g, 4b, d). For participants with AH in the past month, the majority reported hearing voices (63.3%, Fig. 2g)—ranging from unarticulated voices (e.g., screaming, crying, or indistinct speech) to full-sentenced stories (Fig. 2g and Supplementary Fig. 3j). Other frequently reported sounds included music (18.3%) and environmental sounds, e.g., ringtones (17.3%), mechanical human sounds (10.0%), or alarms (4.8%) (Fig. 2g). In a separate multiple-choice question on musical hallucinations (Supplementary Fig. 3e), almost half of the participants (49.6%) reported having experienced AH with a musical content. Among participants with recent VH, shadows (48.8%), people (34.4%), and animals (19.2%) were commonly described (Fig. 4b), as well as less complex images, such as movement (22.2%), light (4.2%), colors (4.5%), or patterns (2.3%) (Fig. 3g). A multiple-choice item on VH content revealed a similar distribution (Supplementary Fig. 3k). Almost half of the participants with TH during the past month reported a sensation of being touched (44.8%; e.g., a hand on their shoulder, being slapped or caressed; Fig. 4b). Other frequently mentioned sensations (Fig. 4b) were formication (23.4%) and tickling or tingling (19.2%). Regarding OH, the most frequently mentioned scents (Fig. 4d) included fire (36.9%), food (14.8%), perfume (10.9%), or flowers (9.6%). Notably, several participants (9.5%) described smelling a nostalgic or familiar scent that was clearly reminiscent of particular childhood memories or relatives. Overall, 149 participants (2.8% of the entire group who reported recent hallucinations) indicated a grief-related component of their experiences, providing examples of hearing (*n* = 30/3086), seeing (*n* = 71/2248), feeling (*n* = 20/2077) or smelling (*n* = 40/1807) a deceased relative or acquaintance. Several participants spontaneously reported a ‘sensed presence’ (*n* = 168/5335), i.e., the clear sensation that someone or something is nearby, without actually being seen or felt^23,24^. Lastly, the open questions allowed us to distinguish other misperceptions, such as illusions, which were reported by small subsamples of participants (AH 2.8%, VH 6.0%, TH 0.3%, OH 0.6%). Although reported by less than 1% of participants, the open questions also provided descriptions that resembled prosopometamorphopsia and other visual distortions, Gedankenlautwerden, musical tinnitus, déjà-vu phenomena, migraine auras, drug-induced imagery, and reperceptive hallucinations.

**Fig. 5:**
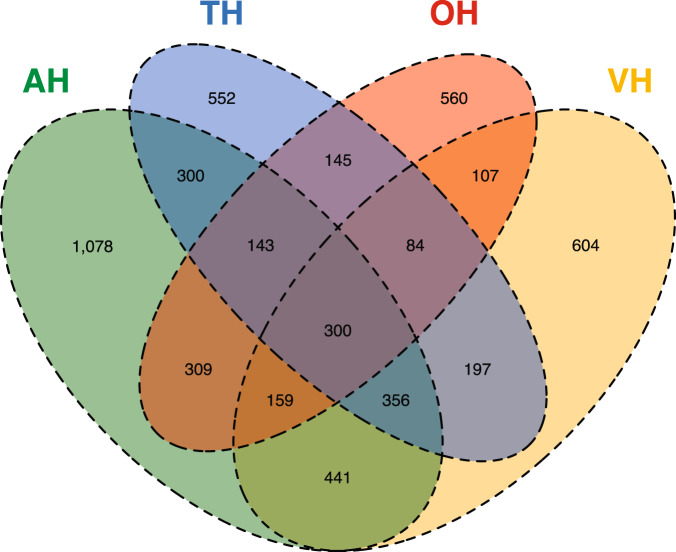
Overlap between four sensory modalities of hallucinations experienced during the past month. Total *n* = 5335. Each colored area represents the sample of participants with hallucinations in the past month in the following sensory modalities: auditory (AH; green), visual (VH; yellow), tactile (TH; blue), and olfactory (OH; red). Overlapping areas indicate participants that experience phenomena in overlapping modalities, the accompanying number of participants indicated within that area. Area sizes are not scaled to proportion.

Figure 5 shows the overlap between the four sensory modalities we studied. Almost half of the participants with hallucinations during the past month (*n* = 2541/5335) had experienced them in at least two sensory modalities; 19.5% in at least three; and 5.6% in all four sensory modalities.

### Association with delusions

Supplementary Figure 6 shows the distribution of delusions (*n* = 6523). Only 454 participants (7.0%) reported being near to fully convinced of the veracity of their delusional ideas (Supplementary Fig. 6a). Delusions of paranoia, reference, and grandeur were reported most frequently (Supplementary Fig. 6b–j).

**Fig. 6:**
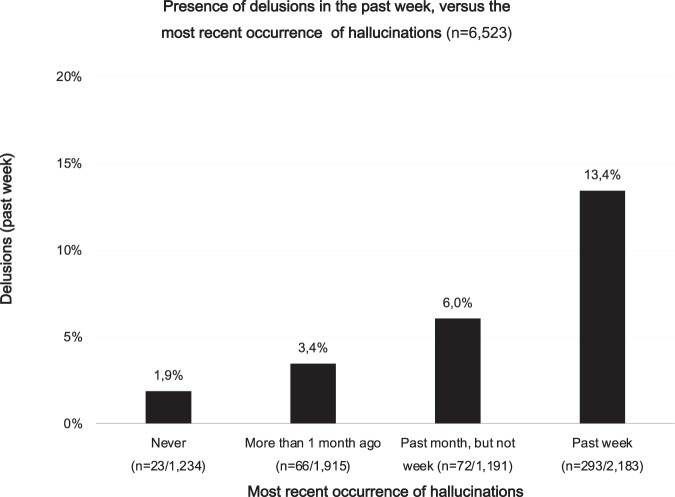
Presence of delusions in the past week, plotted against the most recent occurrence of hallucinations. Total *n* = 6523. The distribution was statistically significant (*χ*^2^ 228.3, *p* < .001, df 3). The presence of hallucinations was categorized as follows: Never, reported having never experienced hallucinations (*n*_delusions_ = 23); more than 1 month ago, reported having ever experienced hallucinations, but not during the past month (*n*_delusions_ = 66); past month but not week, reported having experienced hallucinations during the past month, but not the past week (*n*_delusions_ = 72); past week, reported having experienced hallucinations in the past week (*n*_delusions_ = 293).

Stratification revealed that the percentage of participants with recent delusions was higher in the groups with more recent hallucinations, showing a statistically significant stepwise distribution (*χ*^*2*^ 228.1, *p* < 0.001, df 3), with percentages of delusions up to 13.4% in the group with hallucinations during the past week (Fig. 6).

## Discussion

The current study provides detailed characteristics regarding the content and phenomenology of hallucinations in four sensory modalities, based on self-reports from an online survey of 10,448 individuals from the general population, aged 14 and over. Amongst the wealth of data thus generated, there are several notable findings.

First of all, the reported occurrence of hallucinations and other misperceptions in our study (lifetime 80.1%; past month 51.1%; past week 32.4%) is high in comparison with those reported in prevalence studies of hallucinations, which report prevalence rates averaged between 6 and 15%^2,3,14^. Likely explanations include the broad definition of hallucinations we employed, the low-key way of profiling them (i.e., as very common perceptual phenomena), and the low threshold for reporting on their presence through an online survey.

Secondly, our study provides detailed descriptions of the content of TH, OH, and non-verbal AH (summarized in Figs. 2g, 4b, d), which, in the general population, rarely have been reported before.

In the third place, our results indicate remarkably high percentages of people in the general population showing characteristics of hallucinations that were thus far primarily associated with pathological states. For instance, almost half of the participants experienced hallucinations in more than one sensory modality, a finding that up to now has been predominantly described in patients with schizophrenia, substance abuse, and several neurological disorders^25^. Similarly, a large proportion of participants reported hearing AH with a musical content. Musical hallucinations are typically regarded as rare symptoms in hearing impairment and neurological or psychiatric disorders, or as rare side-effects of the use of medication or illicit substances^26,27^. With our data, however, we cannot rule out that some participants provided false positive responses by addressing similar phenomena, such as musical earworms.

Fourthly, previous studies in the general population have demonstrated an association between the concurrent presence of hallucinations and delusions^22^, a finding that we were able to replicate within our sample. This finding corresponds with neurobiological models of the interaction between hallucinations and delusions in psychosis^8,28,29^. Altogether, our findings suggest that even when applying a very broad definition of hallucinations, they can be closely entwined with the presence of delusions in the general population.

Fifthly, in each sensory modality, we recognized subgroups of participants with more severe phenomenological presentations. From a clinical perspective, these subgroups are of specific interest, since they might be in need of care. Among these subgroups are individuals who experience distress or disfunctioning due to their hallucinations^18,30^, those who experience hallucinations with a high frequency and/or a long duration^18,30^, those who experience hallucinations with a negative content^18,30^, those who have a tendency to comply to dangerous commands issued by their voices^31^, those who report hallucinations associated with previous bothersome experiences^32^, those who present with comorbid delusions^18,30^, and those who report a lack of insight^18,30^. For scientific purposes, these subgroups, as well as those experiencing highly specific hallucination types (e.g., musical hallucinations^5^, auditory-verbal hallucinations^5^, complex visual hallucinations^33^, bereavement hallucinations^34^, or hypnagogia^5,35^) can provide important anchor points for advancing our understanding of the mechanisms underlying hallucinations. Hallucinations are increasingly approached as a phenomenon that occurs across diagnoses, with heterogeneous origins (e.g., increased striatal dopamine, decreased cholinergic activity, social deafferentation, sensory deafferentation)^12^. Indeed, rather than one ‘typical’ phenomenological presentation of hallucinations per disorder, as depicted in Supplementary Table 2, increasing evidence on hallucination phenomenology shows both overlap between diagnostic groups, as well as within-group heterogeneity^18,36–38^. Therefore, the chances of finding common underlying pathways of hallucinations are likely to increase by grouping people with phenomenologically homogeneous presentations (i.e., ‘subtyping’), regardless of diagnosis, and studying and comparing biomarkers within and between these groups (i.e., ‘deep phenotyping’)^12^. We, therefore, recommend to incorporate phenomenological measures in future hallucination research, preferably aided by extensive phenomenological subtyping.

Yet, our most striking finding is the rich phenomenological heterogeneity with which hallucinations presented. Apart from the broad range of content, we observed in all four sensory modalities, each studied phenomenological item showed a highly variable distribution in terms of severity. Following the theory that psychotic symptoms are distributed along a continuum, ranging from isolated, non-clinical symptoms to those occurring in the context of psychotic disorders^2^, our results reflect a similar continuum-like phenomenological spectrum of hallucinations and other misperceptions; with fleeting, neutral, non-distressing, and easily corrigible experiences such as illusions at one side of the spectrum, and persistent, distressing hallucinations that typically present within a clinical context at the other. The heterogeneous phenomenology we observed likely reflects the wide spectrum of severity with which such phenomena can present in the general population. This fits existing neurobiological models of sensory (mis)perception that state that sensory perception consists of afferent stimuli from the actual environment (i.e., bottom-up perception), combined with predictions based on prior expectations about the external environment (i.e., top-down perception). From this perspective, misperceptions are considered common, physiological byproducts of fast, top-down perceptual mechanisms^6,7,9,10,39^, which are, most of the time, easily corrected and transient in nature. Elaborating on these neurobiological models, malfunctioning of the top-down component of the perceptual system at one or more levels may lead to inadequate corrections^7,8^ that we hypothesize to result in misperceptions with a more severe, or even clinical presentation.

From an evolutionary perspective, fast top-down predictions are especially advantageous if they contribute to survival or reproduction. This may explain why the majority of the contents in our sample involved ‘warning signs’ (e.g., hearing threatening voices, sirens, or footsteps; seeing shadows or figures approaching; feeling insects crawling; smelling fire, smoke, or gas), social cues (e.g., hearing telephones ringing, kids crying, or names being called out; seeing familiar people or animals; feeling hands on shoulders; smelling nostalgic scents), and otherwise salient signs (e.g., smelling food). If hallucinations are indeed distributed along a spectrum with other misperceptions and share at least some underlying mechanisms, regardless of their severity, studying misperceptions as a whole (i.e., without any pre-imposed phenomenological definitions, and not excluding illusions or distortions) may provide yet another important avenue to unraveling their pathophysiology.

Overall, our findings provide a more nuanced and less severe perspective on hallucinations and other misperceptions, which are typically depicted in a negative way and often pathologized in Western news media^40^. Likewise, our findings may be helpful to professionals and caregivers for destigmatizing and psycho-educative purposes.

Although using an online survey for data collection provided us with a large and unique dataset that is rich in phenomenological information, this design also has its limitations. Firstly, since data were collected anonymously, it was not possible to directly verify the reliability of study entries. Nevertheless, this degree of anonymity may also have lowered the threshold to participate, and to report openly and honestly about phenomena that would normally be accompanied by embarrassment or fear.

Secondly, as participants drive the inclusion rates entirely, our sample is at risk for sampling and response bias. People with hallucinations may have been more eager to participate, since the topic is likely more salient to them than to others^41^. With regard to the skewed distribution of our sample towards young and female respondents, several personal factors are known to influence response rates, e.g., computer literacy, emotional stability, and conscientiousness^41^. The media through which the study was promoted (Supplementary Fig. 2a, b; Supplementary Table 1) may also have reached specific groups of respondents. Because of this skewed distribution, the positive associations we observed between hallucinations and demographic variables have limited generalizability. The difference between the number of page views and actual participants of the main study suggests that a substantial number of people chose not to participate at all or to prematurely withdraw their participation. Unfortunately, since data of incomplete entries were not recorded, we cannot confirm this, nor objectify reasons for discontinuation. Also, the risk of sampling bias is considerable, which means that the exact percentages of hallucination phenomenology also need to be interpreted with caution.

A third category of limitations involves the QPE. Some QPE-items are at risk for recall bias, including the assessment of lifetime presence of hallucinations and items about the age of hallucination onset. To minimalize this risk for other items, we limited the timeframe of phenomenology assessment to the past month. The QPE-items on delusions and insight in this paper are fully based on self-report. The self-report version of the QPE lacks an additional observant-based rating of these items by a trained interviewer. Since both items are defined by impaired reality testing, self-assessment may have negatively influenced the reliability of these items. Using the QPE, we did not assess some specific hallucination types (e.g., gustatory hallucinations, sexual hallucinations, time distortions), more extensive phenomenological characteristics of TH and OH, nor did we directly screen for the presence of illusions and distortions. A systematic assessment of these other types of misperception in future studies can be advisable. The QPE has no specific item to screen for the presence of pseudohallucinations. This is because the term ‘pseudohallucination’ has been defined in many different ways since its original introduction in 1868 (e.g., hallucinations with intact insight, hallucinations located inside the head, hallucinations in non-psychotic disorders, hallucinations with or without full sensory clarity)^5,42^. The heterogeneity of these operational definitions likely reflects the phenomenological heterogeneity with which hallucinations can present and precludes proper incorporation of the term ‘pseudohallucination’ in a hallucination questionnaire.

Lastly, in contemplation of maintaining the overall accessibility and non-clinical character of the study, it was not possible to incorporate clinical determinants (i.e., medical history, medication use) into this survey. Therefore, we were unable to assess the which extent our included sample overlaps with clinical populations, and in what context the reported hallucinations occurred.

Based on an online survey among over 10,000 participants, this is the first large study to provide an extensive amount of phenomenological information about hallucinations and other misperceptions in four sensory modalities in the general population. Notable findings included the high overlap of hallucinations between two or more sensory modalities, the content of TH, OH, and non-verbal AH, the stepwise increase in delusions in participants with more recent hallucinations, and, most importantly, the heterogeneous phenomenology with which hallucinations and other misperceptions presented, revealing a spectrum-like distribution of severity, and substantial numbers of participants with more severe presentations, such as those with distressing (10.5–16.8%) or debilitating hallucinations (12.7–17.3%). Our findings indicate that misperceptions are both common and phenomenologically diverse, and potentially distributed along a phenomenological continuum in the general population. Apart from the added value for destigmatization and psycho-educative objectives, our observations emphasize the importance of the phenomenological assessment of hallucinations, since they can provide important anchor points for clinical and scientific purposes.

## Methods

### Study design

Using a cross-sectional, observational design, we collected data using an online survey between September 2016 and May 2017. The Medical Research Ethics Committee of the University Medical Center Utrecht exempted this study from full review (local protocol number 16-408/C).

Data collection took place in the Netherlands through a nationally promoted online survey called ‘Zie ik spoken?’ (‘Do I see ghosts?’), which was initiated to promote knowledge of hallucinations in the general population and to open up a public discussion on phenomena still accompanied by stigma and taboo^43,44^. This survey was part of a cooperative project with the national science organization ‘Weekend van de Wetenschap’, (‘Weekend of Science’), an annual scientific event initiated by the Dutch Ministry of Education, Culture, and Science. As such, the study was promoted by national media, including online news websites, TV items, science events, radio and television interviews, online scientific lectures, science events, and articles hosted on scientific websites. Supplementary Fig. 2a, b and Supplementary Table 1 provide an overview of the major events and media entries on the study, and their relation to the total participation rates.

On the project website (https://zieikspoken.nl), we purposefully defined hallucinations in a broad manner, i.e., as perceptions without an external source. During data collection, we presented hallucinations to potential participants as recognizable perceptual phenomena, which can be experienced by anyone. Because of this broad definition, it was likely that some participants, especially in a self-survey, would also report other types of misperception, notably illusions and distortions (Box 1).

### Participants

Any person of 14 years or older and able to understand the Dutch language could participate through the project website. Participants were also recruited at science-related public events, where visitors were invited to take part in the survey at the event site itself and were informed about the study through scientific lectures and interaction with the study team. The local ethical committee considered the study not to fall under the Medical Research Involving Human Subjects Act (WMO). Because of this exemption, the group-based online setting, and the low burden of the study, participants (including underaged participants) provided informed consent via the study website before commencing the study, by checking a statement that their provided data were stored and used for scientific research purposes, anonymously. Participants were then asked to provide information on age, gender, highest level of education, handedness, and whether they were native Dutch speakers, choosing from preset options. Participants could opt out of the study at any point.

### Survey structure

Supplementary Figure 1a shows the survey algorithm. Detailed background information regarding the structure of the online survey is described in Supplementary Note 1.

### Hallucinations

The presence and phenomenology of hallucinations were assessed through self-survey, in four sensory modalities, i.e., auditory (AH), visual (VH), tactile (TH), and olfactory hallucinations (OH), with the aid of the hallucination items of the Questionnaire for Psychotic Experiences (QPE), a 50-item questionnaire specifically designed to assess the presence and phenomenology of hallucinations and delusions across different populations^12,45^. The interview version of the QPE has been validated in several populations^45^. Additionally, a recent study by Kusztritz et al. used the screener questions of the QPE in an online context of self-survey that was similar to ours, demonstrating satisfactory psychometric properties^46^.

An advantage of the QPE is that it applies a very broad definition of hallucinations, without any phenomenological restrictions. In the literature, some definitions of hallucinations incorporate specific conditions regarding phenomenological characteristics such as content, complexity, duration, sleep-wake state, degree of associated distress, or amount of insight into their unreal character^5,18,30^. By refraining from such specifications, the QPE facilitates the assessment of all such phenomenological characteristics in one questionnaire, and hence allows for proper recognition of subgroups of hallucinating participants, based on the reported phenomenology.

The questions from the online hallucination survey are listed in Supplementary Note 2. The survey was largely similar to the hallucination section of the original interviewer-based QPE, with slight adjustments in phrasing in order to directly address the participant.

For each sensory modality, the questionnaire started with a screening item, by means of which participants were asked to report whether they had ever experienced any hallucinations in that sensory modality. If answered affirmatively, participants were then asked whether they had noticed such experiences during the past week, and, if not, during the past month. Hallucination phenomenology was only assessed for hallucinations that were reported within the past week or month. These participants received a series of multiple-choice items about the phenomenology of their hallucinations, i.e., for AH, VH, TH, and OH: frequency, age of onset, and relation to bothersome experiences; for AH, VH: duration, emotional burden, ensuing distress, ensuing disfunctioning, time of the day, perceived location, insight, commands, interaction, and the comorbid presence of illusions. The questionnaire also contained an open question in which participants were asked to provide an example of the content of their experiences (AH, VH, TH, and OH), along with a multiple-choice question regarding the content of voices (AH) or images (VH).

### Delusions

The assessment of delusions was an optional part of the online survey, using the delusion items from the QPE^12,45^. Each of the nine included delusion types (paranoia, reference, guilt, control, religious, grandeur, somatic, Capgras, Cotard) was introduced by a screener question to assess their lifetime presence. If answered affirmatively, the participant was asked to indicate the presence of that thought within the past week, and, if present, how much they were convinced of its truth. Following the definition in the interview-version of the QPE, the reported phenomenon was only considered a delusion if the participant reported being near to fully convinced of their truth. In case of full conviction, participants were asked to answer additional phenomenological questions about the frequency of the phenomenon and any ensuing distress and disfunctioning. When a participant reported to be in doubt or even entirely unconvinced of an idea, the reported phenomenon was not considered delusional in nature, and the questionnaire would continue with the next screener question.

### Data analysis

Supplementary Figure 1b provides an overview of all entries and exclusions in the study databases. Phenomenological details of hallucinations were based on descriptive analyses of the accompanying QPE items. To create a comprehensive overview, we merged phenomenological data from participants who experienced hallucinations in the past week and the past month. The open items of the QPE were used to manually explore and categorize the content of the reported phenomena for each sensory modality. Categories that contained 1% or less of the provided examples were labeled as ‘other’. Delusions were categorized as either absent or present; presence was defined as ‘having occurred during the past week, with near to full conviction’. Demographic characteristics and delusions were compared between four distinct subgroups, categorized based on the most recent occurrence of hallucinations: never experienced (‘never’); experienced, but not during the past month (‘more than 1 month ago’); experienced during the past month, but not during the past week (‘past month but not week’), and experienced during the past week (‘past week’). We used Pearson’s chi-square for categorical data, and, depending on the normality of their distribution (based on Kolmogorov–Smirnov tests and visual inspection of the raw data distribution), a one-way ANOVA or Kruskal–Wallis-test for continuous data. Analyses were performed using IBM SPSS Statistics version 22.0.

### Reporting summary

Further information on research design is available in the Nature Research Reporting Summary linked to this article.

## Supplementary information

REPORTING SUMMARY

Supplemental material

## Data Availability

The data that support the findings of this study are available on request from the corresponding author M.M.J.L. The data are not publicly available, due to them containing information that could compromise a research participant’s privacy or consent.
