# Supplementary material for: Occurrence and phenomenology of hallucinations in the general population: A large online survey
Source: Schizophrenia (Heidelb). 2022 Apr 23;8(1):41. doi: 10.1038/s41537-022-00229-9 (PMC9261095; doi:10.1038/s41537-022-00229-9)
Supplement: Supplementary file 1 — REPORTING SUMMARY [file 41537_2022_229_MOESM1_ESM.pdf]

nature research

Corresponding author(s): Mascha Linszen

Last updated by author(s): May 28, 2021

Reporting Summary

Nature Research wishes to improve the reproducibility of the work that we publish. This form provides structure for consistency and transparency in reporting. For further information on Nature Research policies, see our Editorial Policies and the Editorial Policy Checklist.

Statistics

For all statistical analyses, confirm that the following items are present in the figure legend, table legend, main text, or Methods section.

n/a	Confirmed	
☐	☒	The exact sample size (*n*) for each experimental group/condition, given as a discrete number and unit of measurement
☐	☒	A statement on whether measurements were taken from distinct samples or whether the same sample was measured repeatedly
☐	☒	The statistical test(s) used AND whether they are one- or two-sided<br>*Only common tests should be described solely by name; describe more complex techniques in the Methods section.*
☒	☐	A description of all covariates tested
☐	☒	A description of any assumptions or corrections, such as tests of normality and adjustment for multiple comparisons
☐	☒	A full description of the statistical parameters including central tendency (e.g. means) or other basic estimates (e.g. regression coefficient) AND variation (e.g. standard deviation) or associated estimates of uncertainty (e.g. confidence intervals)
☐	☒	For null hypothesis testing, the test statistic (e.g. *F*, *t*, *r*) with confidence intervals, effect sizes, degrees of freedom and *P* value noted<br>*Give P values as exact values whenever suitable.*
☒	☐	For Bayesian analysis, information on the choice of priors and Markov chain Monte Carlo settings
☒	☐	For hierarchical and complex designs, identification of the appropriate level for tests and full reporting of outcomes
☒	☐	Estimates of effect sizes (e.g. Cohen's *d*, Pearson's *r*), indicating how they were calculated

Our web collection on statistics for biologists contains articles on many of the points above.

Software and code

Policy information about availability of computer code

Data collection	The website and survey were built using WordPress version 4.6.10. The survey itself was designed using the most recent updates of the plugin Gravity Forms. Raw data was downloaded from the online study database in the form of an .csv-file. Data was imported in IBM SPSS Statistics via Microsoft Excel (version Microsoft Office Professional Plus 2016 for Windows).
Data analysis	Analyses were performed using IBM SPSS Statistics version 22.0.

For manuscripts utilizing custom algorithms or software that are central to the research but not yet described in published literature, software must be made available to editors and reviewers. We strongly encourage code deposition in a community repository (e.g. GitHub). See the Nature Research guidelines for submitting code & software for further information.

Data

Policy information about availability of data

All manuscripts must include a data availability statement. This statement should provide the following information, where applicable:

- Accession codes, unique identifiers, or web links for publicly available datasets
- A list of figures that have associated raw data
- A description of any restrictions on data availability

The data that support the findings of this study are available on request from the corresponding author. The data are not publicly available, since they contain information that could compromise a research participant’s privacy or consent.

Field-specific reporting

Please select the one below that is the best fit for your research. If you are not sure, read the appropriate sections before making your selection.

☐ Life sciences ☒ Behavioural & social sciences ☐ Ecological, evolutionary & environmental sciences

For a reference copy of the document with all sections, see nature.com/documents/nr-reporting-summary-flat.pdf

Behavioural & social sciences study design

All studies must disclose on these points even when the disclosure is negative.

Study description	Cross-sectional, observational study, that uses an online survey to obtain quantitative data on hallucination occurrence and phenomenology and the presence of delusions, as well as some qualitative data on hallucination content.
Research sample	The target population of this survey was the general Dutch population aged 14 and over. Respondents were reached via several national media through which the study was promoted. The sample is relatively skewed towards highly educated, young, female participants. Although this limits the generalizability of our results, it is likely that our choice of design has facilitated a degree of anonymity that may have lowered the threshold to participate and to report openly and honestly about phenomena that would normally be accompanied by embarrassment or fear.
Sampling strategy	Random sampling procedure, based on complete responses to an online survey. No predetermined sample size was applied. The study start and end dates were largely based on the scheduled promotional events. Additionally, after the last scheduled promotional event, the study was eventually stopped when the weekly added amount of participants had been 30 respondents per week or less for over two months.
Data collection	The website and survey were built using WordPress. The survey itself was designed using the plugin Gravity Forms. Each participant could access the study and participate using a web browser on their mobile phones, iPad or computer. The researcher was not present during participation. Only complete responses were saved as an entry in the study database.
Timing	Start date of data collection: September 26, 2016. End date of data collection: May 23, 2017.
Data exclusions	Entries were excluded in case of invalidity of data: duplicate entries (n=727), test entries (n=5) and entries with unlikely outliers (n=2). Additionally, we excluded 419 entries with a reported age younger than 14 years.
Non-participation	Within the timeframe of data collection, we recorded 23,577 page views. In comparison with the total amount of valid responses in the study database (n=10,867), there have been 12,710 page views without a complete response. These additional page views suggest that a substantial number of people chose not to participate at all or to prematurely withdraw their participation, but since data of incomplete entries were not recorded, we cannot confirm this, nor objectify reasons for discontinuation.
Randomization	This study had no randomization procedure since randomization was not part of the study design.

Reporting for specific materials, systems and methods

We require information from authors about some types of materials, experimental systems and methods used in many studies. Here, indicate whether each material, system or method listed is relevant to your study. If you are not sure if a list item applies to your research, read the appropriate section before selecting a response.

Materials & experimental systems

n/a	Involved in the study	
☒	☐	Antibodies
☒	☐	Eukaryotic cell lines
☒	☐	Palaeontology and archaeology
☒	☐	Animals and other organisms
☐	☒	Human research participants
☒	☐	Clinical data
☒	☐	Dual use research of concern

Methods

n/a	Involved in the study	
☒	☐	ChIP-seq
☒	☐	Flow cytometry
☒	☐	MRI-based neuroimaging

Human research participants

Policy information about studies involving human research participants

Population characteristics	See above
Recruitment	See above; recruitment took place through promotion of the online survey through several national media. Since anyone

Recruitment	aged 14 and above could participate in the study, there is a risk of sampling and response bias. As our data are skewed towards young and female respondents, the positive associations we observed between hallucinations and demographic variables have limited generalizability. This also means means that the exact percentages of hallucination phenomenology need to be interpreted with caution.
Ethics oversight	Local Medical Research Ethics Committee of the University Medical Center Utrecht, the Netherlands

Note that full information on the approval of the study protocol must also be provided in the manuscript.
