# Supplementary material for: Occurrence and phenomenology of hallucinations in the general population: A large online survey
Source: Schizophrenia (Heidelb). 2022 Apr 23;8(1):41. doi: 10.1038/s41537-022-00229-9 (PMC9261095; doi:10.1038/s41537-022-00229-9)
Supplement: Supplementary file 2 — Supplemental material [file 41537_2022_229_MOESM2_ESM.pdf]

Supplementary material

Material	Page
**Supplementary Figure 1a**<br>*Flowchart of the applied algorithm of the online survey*	2
**Supplementary Figure 1b**<br>*Flowchart of participation rates*	3
**Supplementary Note 1**<br>*Detailed background information of the survey structure*	4
**Supplementary Figure 2a**<br>*Graphical representation of study participants*	6
**Supplementary Figure 2b**<br>*Graphical representation of study participants (initial phase)*	7
**Supplementary Table 1**<br>*Listed overview of promotional events*	8
**Supplementary Figure 3**<br>*Additional phenomenological details of auditory hallucinations*	10
**Supplementary Figure 4**<br>*Additional phenomenological details of visual hallucinations*	11
**Supplementary Figure 5**<br>*Additional phenomenological details of tactile and olfactory hallucinations*	12
**Supplementary Figure 6**<br>*Distribution of delusions*	13
**Supplementary Table 2**<br>*‘Typical’ phenomenology of hallucinations in various disorders*	14
**Supplementary Note 2**<br>*Questionnaire Psychotic Experiences (online version, in Dutch)*	16

Supplementary Figure 1a

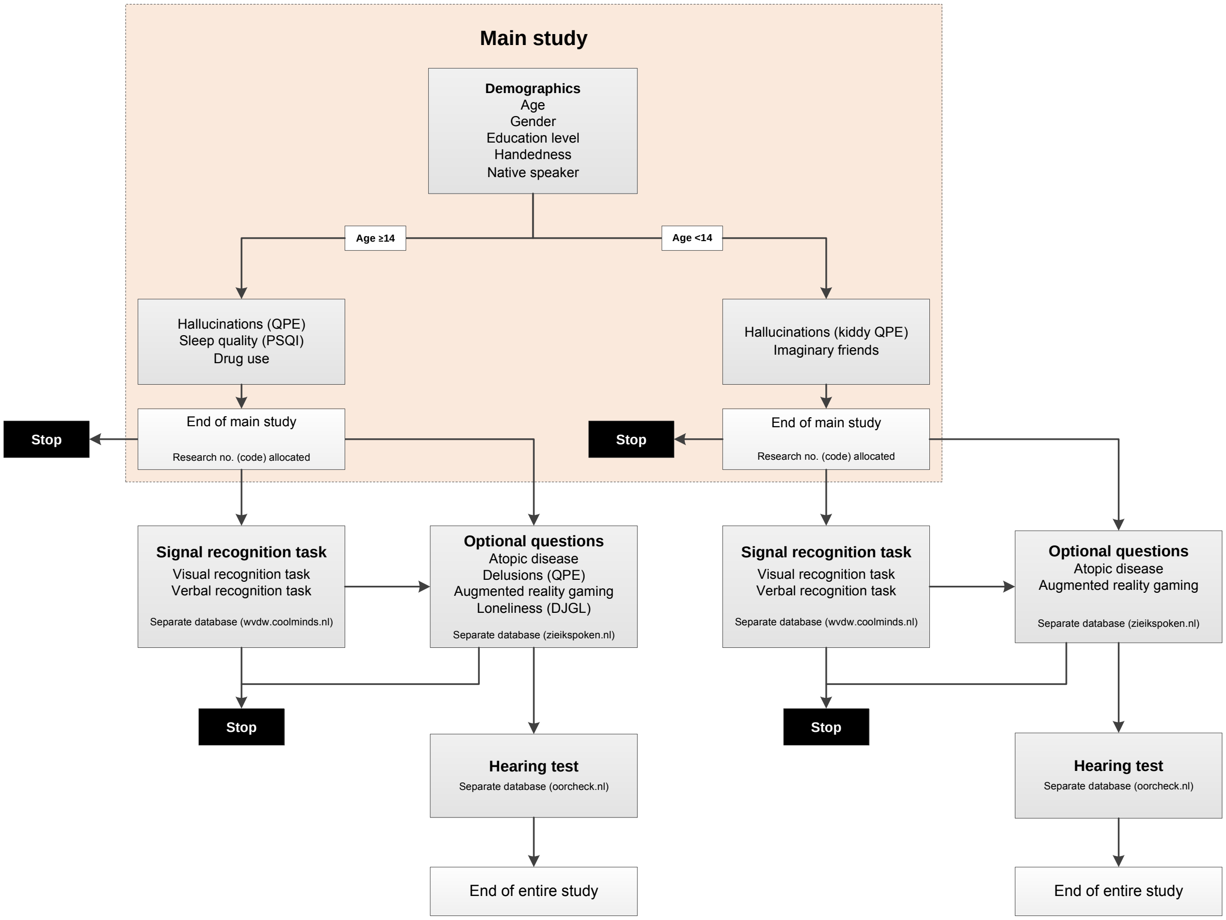

Supplementary Figure 1a. Flowchart of the applied algorithm of the online survey. The fuchsia-colored square marks the content of the main study. The distinction between participants over or under 14 was established on September 30, 2016, four days after the official launch of the survey.

Supplementary Figure 1b

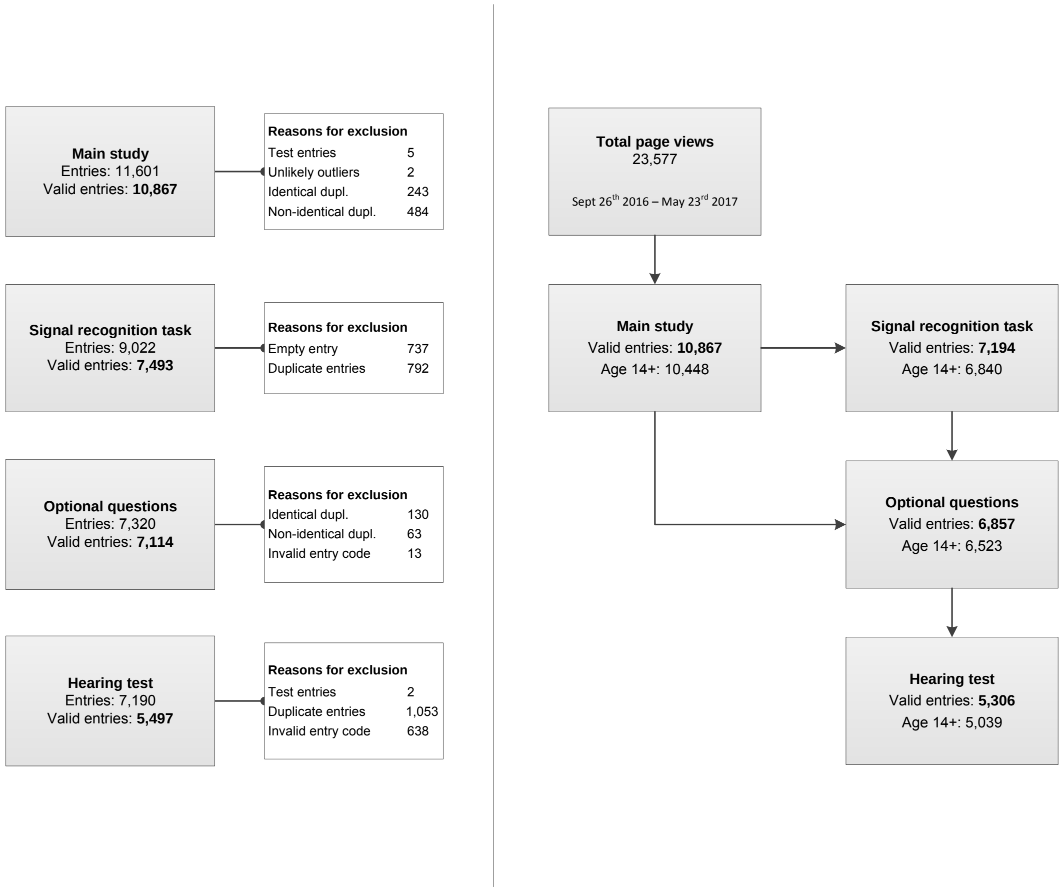

Supplementary Figure 1b. Left: Participation rates of each separate database before and after data cleaning, including reasons for exclusion. Dupl, duplicate entry.
Right: Flowcharts of participants after data cleaning and merging of separate databases, providing the remaining valid entries after merging of databases, both overall and after selection of participants aged 14 or over.

Supplementary Note 1

Overall survey structure

Overall, the survey consisted of four parts (referred to as 1. main study, 2. signal recognition task, 3. optional questions, 4. hearing test), each of which was connected with a separate database. Two parts of the total survey (main study; optional questions) were part of the internal structure of the study website. The two other parts (signal recognition task; hearing test) relied on data collection from external online sources. All data from completed survey entries were saved within secured online databases that could only be accessed through involved study personnel. Incomplete entries were not registered. Supplementary figure 1a shows the complete algorithm that a participant was able to follow once entering the survey.

As shown in supplementary figure 1a, the first step for every participant was to enter the main study, and to provide informed consent and several demographic characteristics. Participants younger than 14 years were directed to the 'kiddy' part of the main study, which contained questionnaires on hallucination-like experiences and imaginary friends, which were specifically designed to suit younger participants and are not included in the current study. Participants aged 14 and over were redirected to complete the complete main part of the study, consisting of questionnaires on hallucination-like experiences, sleep, and recreational drug use.

At the end of the main study the participant was alerted about completing the first part of the survey, and discretely allocated with an entry-specific research number. This concluding webpage also contained separate links inviting participants to continue their participation in two subsequent parts of the study: the signal recognition task (accessed through https://wvdw.coolminds.nl) and the optional questions. Once accessed, the allocated research number was automatically entered in both databases, invisible to the participant, so that the outcomes of both surveys could be linked with data from the main one.

Within the optional questions section, participants were again questioned whether they were aged 14 and over or younger, on which the arrangement of the remaining questionnaires was based. Participants aged 14 and over were directed to questionnaires about atopic disease, delusion-like experiences, loneliness and augmented reality gaming. Participants younger than 14 were only presented with questionnaires on atopic disease and augmented reality gaming.

Finally, all participants that completed the optional question part of the survey were presented with a link to the final part: the hearing test, which could be accessed through an external website (https://ahersenonderzoek.oorcheck.nl and https://hersenonderzoek.oorcheck.nl). Once redirected, participants were asked to manually enter their previously allocated research number to ensure the link with previous data. Completion of the hearing test indicated the end of the entire survey.

Data collection for this study started on September 26, 2016, during the official press launch of the national scientific event Weekend van de Wetenschap 2016, and was discontinued on May 23, 2017, after which data from each of the four databases were collected. Before merging the additional datasets with the database from the main study, each dataset was checked for duplicate and invalid entries, which were then removed. Merging of all databases took place in SPSS statistics version 24, by connecting all entries through the entered research number.

Supplementary Figure 2a

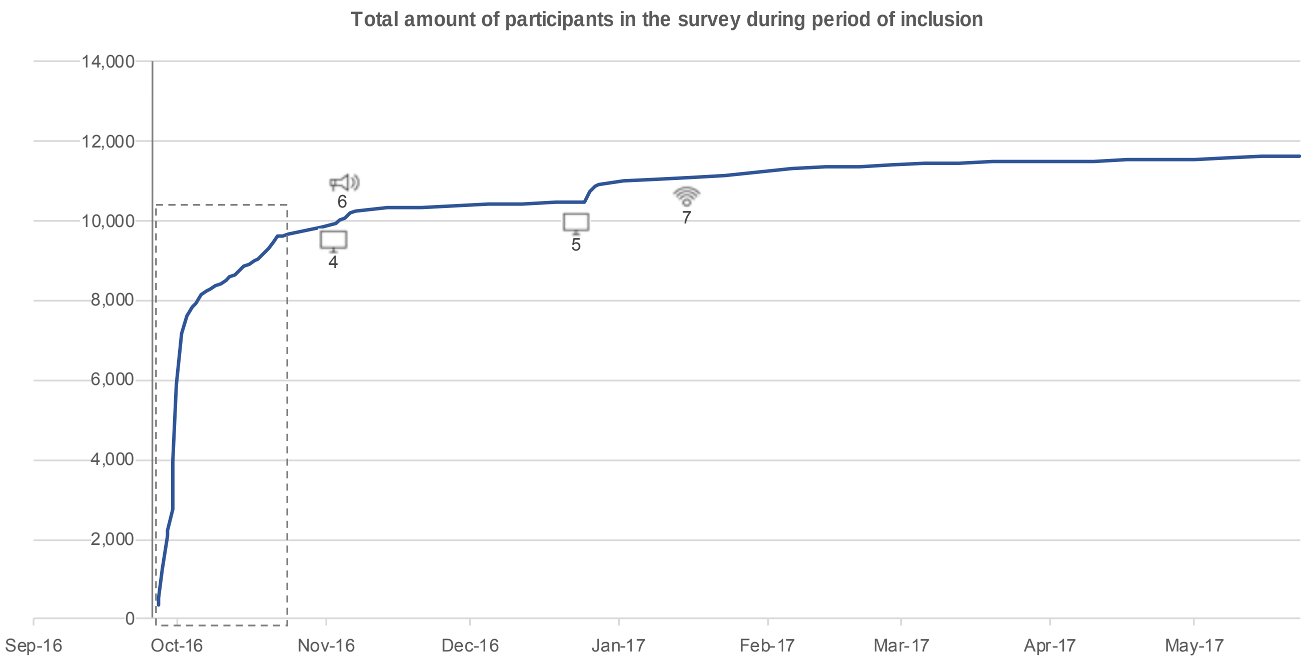

Supplementary Figure 2a. Graphical representation of the total amount of study participants during inclusion phase (September 26, 2016 – May 23, 2017). The dashed rectangle represents the initial promotion phase (September 26, 2016 – October 24, 2016); see supplementary figure 1b for a more detailed overview. Promotional events are indicated with an icon and number; corresponding events are listed in supplementary table 1.

Supplementary Figure 2b

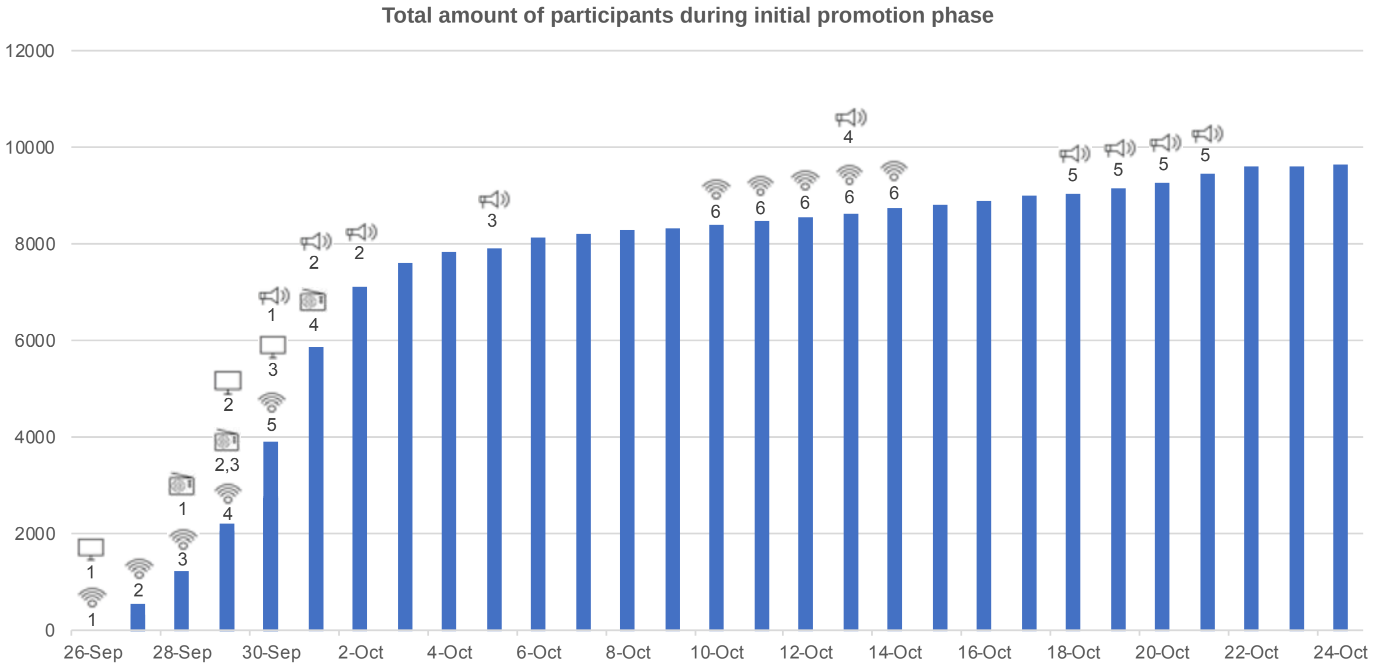

Supplementary Figure 2b. Graphical representation of the total amount of study participants during the initial promotion phase (September 26, 2016 – October 24, 2016). Promotional events are indicated with an icon and number; corresponding events are listed in supplementary table 1.

Supplementary Table 1

Supplementary Table 1. Listed overview of promotional events, as shown in supplementary figures 2ab.

Online

1. September 26, 2016. Official press release and launch of website 'Zie ik Spoken' as the annual national population study by 'Weekend van de Wetenschap' (link, in Dutch).
2. September 27, 2016. National news website Nu.nl, article, 'Start of nationwide study on hallucinations' (link, in Dutch)
3. September 28, 2016. National science website NEMO Kennislink. Article, 'Do I see ghosts? Large scale study on hallucinations'. (link, in Dutch)
4. September 29, 2016. National news website NOS op 3, article and interview, 'Hallucinations are mistakenly stigmatized' (link, in Dutch)
5. September 30, 2016. National news website and TV-program 'RTL nieuws: Editie NL', interview and special "Everyone hallucinates, and some people see a dead body every hour". (link, in Dutch)
6. October 10 – 14, 2016. Online scientific platform 'De Universiteit van Nederland', special lectures on hallucinations by Iris Sommer, Jan Dirk Blom and Mascha Linszen. (link, in Dutch)
7. January 18, 2017. Second official press release by 'Weekend van de Wetenschap', based on interim results. (link, in Dutch).

Television

1. September 26, 2016. National TV show 'RTL Late Night', interview Iris Sommer, 'How do you score on these hallucination tests?' (link, in Dutch)
2. September 29, 2016. Dutch scientific TV-program 'Met de kennis van nu', special "Everyone hallucinates". (link, in Dutch)
3. September 30, 2016. National news website and TV-program 'RTL nieuws: Editie NL', interview and special "Everyone hallucinates, and some people see a dead body every hour". (link, in Dutch)
4. November 3, 2016. National TV show 'De Wereld Draait Door', interview Iris Sommer.
5. December 25, 2016. National Science Quiz, TV program (de Nationale

Wetenschapsquiz), participation by Iris Sommer (link, in Dutch)

Radio

1. September 28, 2016. National radio station Radio 2, program ‘de Roodshow’, interview Iris Sommer (link, in Dutch)
2. September 29, 2016. National radio station 3FM, program ‘Giel’, interview Iris Sommer (link, in Dutch)
3. September 29, 2016. National radio station Radio 1, ‘Met het oog op morgen’, interview Iris Sommer (link, in Dutch)
4. October 1, 2016. National radio station Radio 2, program ‘Echt Jasper’, interview Iris Sommer (link, in Dutch).

Events

1. September 30, 2016. Public event ‘Betweter festival’, arts and science festival, Tivoli Vredenburg, Utrecht. Lecture ‘the Hallucinating Human Being’ and local promotion (link, in Dutch).
2. October 1 and 2, 2016. National public science event ‘Weekend van de wetenschap’. Local promotion at NEMO science museum, Amsterdam.
3. October 5, 2016. Public science event ‘Tilburg Night University’. Local promotion at Tilburg University, Tilburg. (link)
4. October 13, 2016. Public event of the Dutch Brain Society (‘Hersenstichting’). Local promotion at Beatrix theater, Utrecht.
5. October 18 – 21, 2016. Public science event during the autumn holidays. Local promotion at the university museum, Utrecht.
6. November 5, 2016. Public event ‘Museum night Amsterdam’. Local promotion at NEMO science museum, Amsterdam.

Supplementary Figure 3

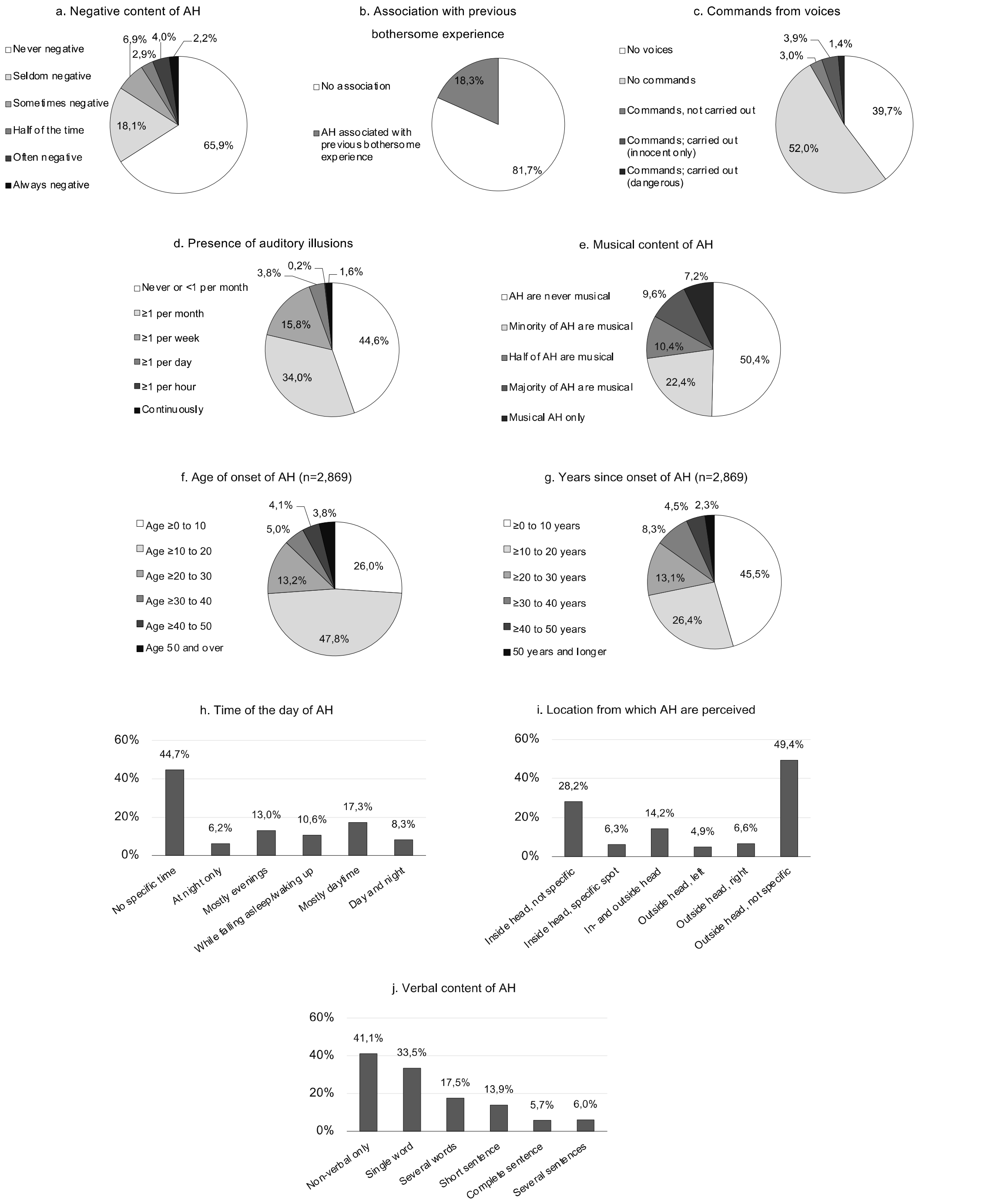

Supplementary Figure 3. Additional phenomenological items of auditory hallucinations (AH) (n=3,086), obtained through QPE multiple choice items with one answer possibility (Supp.Fig.3a-h) or multiple answer possibilities (Supp.Fig.3i, 3j; total percentage exceeds 100%). The total amount of surveyed participants is 3,086 for every item, unless specified otherwise. The age of onset (Figure 1d) has been computed by subtracting the answers from figure 2c from the participants' age. Due to an inaccurately programmed answer in the online version of QPE item 1.7 ("Repetition of AH"), this item has been excluded from current analysis in order to retain overall validity. In Figure 1o, a small sample of participants (n=55; 1.8%) provided answer combinations that appeared contradictory (i.e., both a specific and non-specific location). Similarly, in Figure 1p, 332 participants (10.8%) provided potentially contradictory answer combinations (i.e., 'non-verbal only' with one or more verbal answers). In both items, these entries have been maintained to provide the most accurate representation of the obtained data.

Supplementary Figure 4

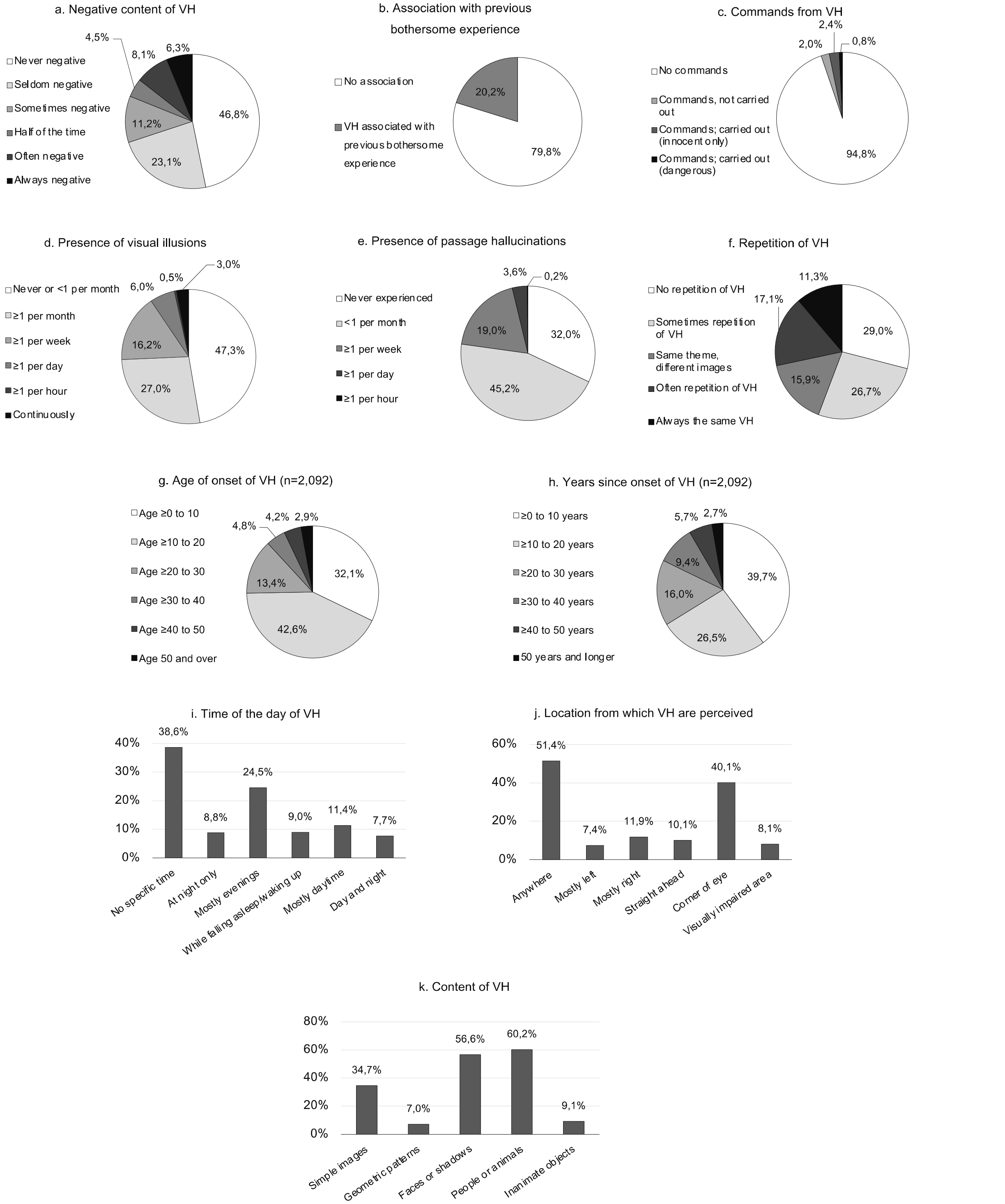

Supplementary Figure 4. Additional phenomenological items on visual hallucinations (VH) (n=2,248), obtained through multiple choice items with one answer possibility (Supp.Fig. 4a-i) and multiple answer possibilities (Supp. Fig. 4j, 4k; total percentage exceeds 100%). The total amount of surveyed participants is 2,248 for every item, unless specified otherwise. The age of onset (Supp.Fig. 4h) has been computed by subtracting the answers from Supp.Fig. 4g from the participants' age. In Supp.Fig. 4j, a sample of participants (n=267; 11.9%) provided answer combinations that appeared contradictory (e.g., mostly left and mostly right). These entries have been maintained to provide the most accurate representation of the obtained data.

Supplementary Figure 5

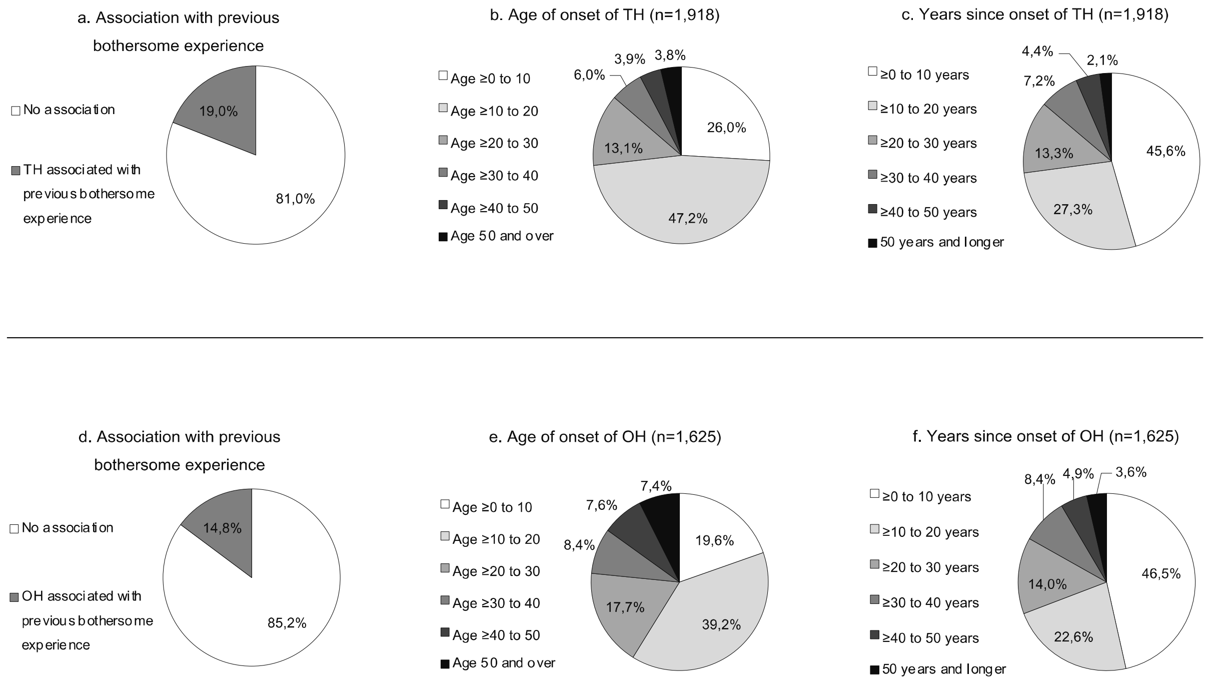

Supplementary Figure 5. Additional phenomenological items on tactile hallucinations (TH) (n=2,077) (Supp.Fig. 5a-c) and olfactory hallucinations (OH) (n=1,807) (Supp.Fig. 5d-f), obtained through QPE multiple choice items with one answer possibility. The age of onset (Supp.Fig. 5c (TH); 5f (OH)) has been computed by subtracting the answers from Supp.Fig. 5b (TH) and Supp. Fig. 5e (OH) from the participants' age.

Supplementary Figure 6

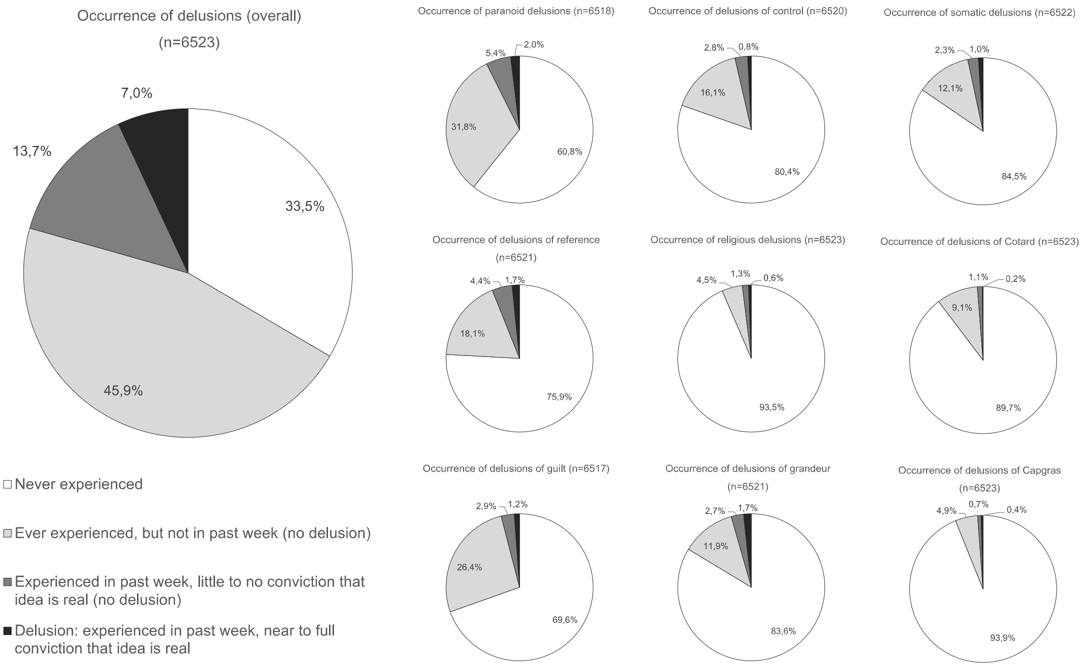

Supplementary Figure 6. Occurrence of delusions based on accompanying QPE entries, in all participants that completed the second part of the survey and were aged 14 and over (n=6523). An entry was only considered a delusion if reported with near to full conviction about the real character of the reported belief. The 9 small figures on the right reflect the distribution of answer per delusion-type. Data is missing due to invalid entries in 0-6 participants, depending on delusion-type. The large figure on the left summarizes entries on nine different delusion-types altogether, by showing the overall distribution of delusions in the entire sample.

Supplementary Table 2.

Supplementary Table 2. Phenomelogical features of hallucinations that are considered 'typical' for each of the mentioned diagnostic categories.

	**'Typical' phenomenological presentation**							
**Diagnostic category**	**Modality**	**Content**	**Emotional valence**	**Frequency**	**Induced distress**	**Insight in unreal character**	**Delusions**	**References**
Schizophrenia spectrum disorder	Auditory (Sometimes: VH, TH, OH)	Voices (commands, commentary, Gedankenlautwerden)	Negative	Daily to continuous	Severe	Partially to fully absent	Present	Schutte et al., 2020; Daalman et al., 2012; Blom and Sommer, 2010
Drug-induced psychosis	Tactile	Formication	-	Daily to continuous	Severe	Partially to fully absent	Present	McKetin et al., 2017
Parkinson's disease	Visual (Sometimes: multimodal)	People, animals	Neutral	Weekly to daily	Little to moderate	Intact	Sometimes present	Burghaus et al., 2012; Urwyler et al., 2016
Dementia with Lewy Bodies	Visual (Sometimes: multimodal)	People, animals	Neutral	Weekly to daily	Little to moderate	Partially intact	Sometimes present	Burghaus et al., 2012; Urwyler et al., 2016
Visual impairment (Charles Bonnet Syndrome)	Visual	Simple geometric images, people, animals	Neutral	Monthly to weekly	Little to moderate	Intact	Absent	Teunisse et al., 1996; Urwyler et al., 2016
Hearing impairment	Auditory	Music, voices, acoasms	Neutral	Monthly to weekly	Little to moderate	Intact	Absent	Teunisse and Olde Rikkert, 2012; Linszen et al., 2018; Schutte et al., 2020
Borderline personality disorder	Auditory (Sometimes: VH, TH, OH)	Voices	Negative	Daily to continuous	Severe	Partially to fully absent	Transient paranoid ideas	Slotema et al. 2019; Kingdon et al., 2010
Delirium	Visual	People, animals	-	-	-	Absent	Sometimes present	Cutting, 1987; Leentjens et al., 2008
Phantosmia	Olfactory	Smoky, burnt	-	Monthly to weekly	-	Intact	Absent	Sjölund et al., 2017

List of references

Blom, J. D., & Sommer, I. E. C. (2010). Auditory Hallucinations: nomenclature and classifications. Cognitive and Behavioural Neurology, 23(1), 55–62.

Burghaus, L., Eggers, C., Timmermann, L., Fink, G. R., & Diederich, N. J. (2012). Hallucinations in Neurodegenerative Diseases. CNS Neuroscience and Therapeutics, 18(2), 149–159.

Cutting, J. (1987). The phenomenology of acute organic psychosis. Comparison with acute schizophrenia. British Journal of Psychiatry, *151*(SEPT.), 324–332.

Daalman, K., Boks, M. P. M., Diederen, K. M. J., De Weijer, A. D., Blom, J. D., Kahn, R. S., & Sommer, I. E. C. (2011). The same or different? A phenomenological

comparison of auditory verbal hallucinations in healthy and psychotic individuals. Journal of Clinical Psychiatry, 72(3), 320–325.

Kingdon, D. G., Ashcroft, K., Bhandari, B., Gleeson, S., Warikoo, N., Symons, M., Tyler, L., Lucas, E., Mahendra, R., Ghosh, S., Mason, A., Badrakalimuthu, R., Hepworth, C., Read, J., Mehta, R. (2010). Schizophrenia and borderline personality disorder: Similarities and differences in the experience of auditory hallucinations, paranoia, and childhood trauma. Journal of Nervous and Mental Disease, 198(6), 399–403.

Leentjens, A. F. G., Schieveld, J. N. M., Leonard, M., Lousberg, R., Verhey, F. R. J., & Meagher, D. J. (2008). A comparison of the phenomenology of pediatric, adult, and geriatric delirium. Journal of Psychosomatic Research, 64(2), 219–223.

Linszen, M. M. J., Van Zanten, G. A., Teunisse, R. J., Brouwer, R. M., Scheltens, P., & Sommer, I. E. (2019). Auditory hallucinations in adults with hearing impairment: A large prevalence study. Psychological Medicine, 49(1).

McKetin, R., Baker, A. L., Dawe, S., Voce, A., & Lubman, D. I. (2017). Differences in the symptom profile of methamphetamine-related psychosis and primary psychotic disorders. Psychiatry Research, 251(February), 349–354.

Schutte, M. J. L., Linszen, M. M. J., Marschall, T. M., ffytche, D. H., Koops, S., van Dellen, E., Heringa, S.M., Slooter, A.J.C., Teunisse, R., van den Heuvel, O.A., Lemstra, A.W., Foncke, E.M.J., Slotema, C.W., de Jong, J., Rossell, S.L., Sommer, I. E. C. (2020). Hallucinations and other psychotic experiences across diagnoses: A comparison of phenomenological features. Psychiatry Research, 292(July), 113314.

Sjölund, S., Larsson, M., Olofsson, J. K., Seubert, J., & Laukka, E. J. (2017). Phantom smells: Prevalence and correlates in a population-based sample of older adults. Chemical Senses, 42(4), 309–318.

Slotema, C. W., Bayrak, H., Linszen, M. M. J., Deen, M., & Sommer, I. E. C. (2019). Hallucinations in patients with borderline personality disorder: characteristics, severity, and relationship with schizotypy and loneliness. Acta Psychiatrica Scandinavica, 139(5), 434–442.

Teunisse, R. J., Cruysberg, J. R., Hoefnagels, W. H., Verbeek, A. L., & Zitman, F. G. (1996). Visual hallucinations in psychologically normal people: Charles Bonnet’s syndrome. Lancet, 347(9004), 794–797.

Teunisse, R. J., & Olde Rikkert, M. G. M. (2012). Prevalence of musical hallucinations in patients referred for audiometric testing. American Journal of Geriatric Psychiatry, 20(12), 1075–1077.

Supplementary Note 2
Applied online version of the QPE. In Dutch, translation upon request.

Introductietekst

Dit is een vragenlijst over speciale ervaringen of ideeën die je mogelijk hebt gehad. Voorbeelden zijn het horen van stemmen, het zien van beelden en wantrouwig of in de war zijn. Deze ervaringen komen niet alleen voor bij mensen met verschillende diagnoses, maar ook bij gezonde mensen. Dit interview gaat over de aanwezigheid van deze ervaringen of ideeën. Probeer het antwoord te geven dat het beste bij je past. Niet alle vragen hoeven op jou persoonlijk van te toepassing te zijn.

1. Het komt wel eens voor dat men iemand hoort spreken, terwijl er niemand lijkt te zijn. Ook kunnen geluiden of muziek worden gehoord, terwijl het niet duidelijk is waar dat vandaan komt. Heb jij wel eens zulke stemmen, muziek of andere geluiden gehoord?

0: nee
1: ja

Zo ja, heb je dit in de afgelopen week nog ervaren?

0: nee
1: ja

⇨ Indien deze vraag met 'ja' wordt item: aanvullen met item 1.1 t/m 1.15 uit de appendix

Zo nee, heb je dit in de afgelopen maand nog ervaren?

0: nee
1: ja

⇨ Indien deze vraag met 'ja' wordt item: aanvullen met item 1.1 t/m 1.15 uit de appendix. De items over 'de afgelopen week' hebben in dat geval betrekking op de afgelopen maand, en zijn ook als dusdanig geprogrammeerd.

2. Het komt wel eens voor dat men een persoon, dier of ding ziet, die anderen niet kunnen zien. Sommige mensen zien soms een schaduw of schim. Heb jij wel eens zulke dingen, personen of beelden gezien?

0: nee
1: ja

Zo ja, heb je dit in de afgelopen week nog ervaren?

0: nee
1: ja

⇨ Indien deze vraag met ‘ja’ wordt item: aanvullen met item 2.1 t/m 2.15 uit de appendix

Zo nee, heb je dit in de afgelopen maand nog ervaren?

0: nee

1: ja

⇨ Indien deze vraag met ‘ja’ wordt item: aanvullen met item 2.1 t/m 2.15 uit de appendix. De items over ‘de afgelopen week’ hebben in dat geval betrekking op de afgelopen maand, en zijn ook als dusdanig geprogrammeerd.

3. Sommige mensen hebben wel eens het gevoel aangeraakt te worden, of een hand op de schouder te voelen terwijl er niemand in de buurt is. Een ander voorbeeld is iemand die beestjes onder zijn huid voelt kriebelen die er niet zijn. Heb jij zoiets wel eens ervaren?

0: nee

1: ja

Zo ja, heb je dit in de afgelopen week nog ervaren?

0: nee

1: ja

⇨ Indien deze vraag met ‘ja’ wordt item: aanvullen met item 3.1 en 3.2 uit de appendix

Zo nee, heb je dit in de afgelopen maand nog ervaren?

0: nee

1: ja

⇨ Indien deze vraag met ‘ja’ wordt item: aanvullen met item 3.1 en 3.2 uit de appendix.

4. Sommige mensen ruiken wel eens dingen die er niet zijn. Bijvoorbeeld een brandlucht, terwijl er geen brand is. Een ander voorbeeld is dat iemand bloemen ruikt, terwijl er geen bloemen in de buurt zijn. Heb jij zoiets wel eens ervaren?

0: nee

1: ja

Zo ja, heb je dit in de afgelopen week nog ervaren?

0: nee

1: ja

⇨ Indien deze vraag met ‘ja’ wordt item: aanvullen met item 4.1 en 4.2 uit de appendix

Zo nee, heb je dit in de afgelopen maand nog ervaren?

0: nee

1: ja

⇨ Indien deze vraag met ‘ja’ wordt item: aanvullen met item 4.1 en 4.2 uit de appendix.

Appendix 1.1: additionele vragen indien item 1 positief is beantwoord.

1.1. **Hoe vaak hoor je de stemmen of geluiden?**

0: Minder dan een keer per maand
1: Minimaal eens per maand
2: Minimaal eens per week
3: Minimaal eens per dag
4: Minimaal eens per uur
5: (Bijna) altijd

1.2. **a. Wat hoor je precies? Kan je een voorbeeld geven?**

……………………………………………………………………………………………………

……………………………………………………………………………………………………

……………………………………………………………………………………………………

b. Sinds wanneer hoor je zulke stemmen of geluiden? Op welke leeftijd is dit begonnen?

……………………………………………………………………………………………………

……………………………………………………………………………………………………

c. Bij sommige mensen houdt dit verband met eerdere vervelende gebeurtenissen.

Is dit bij jou het geval? Ja/Nee

1.3. **Wanneer je de stemmen of geluiden in de afgelopen week hoorde, hoe lang duurde dit toen?**

0: Heel kort; slechts een ogenblik
1: Een paar seconden
2: Een minuut of hooguit enkele minuten
3: 10 minuten tot een uur
4: Een uur tot enkele uren
5: (Bijna) altijd

1.4. **Sommige mensen horen stemmen of geluiden met een negatieve inhoud, waaronder kritiek en vijandigheid. Welk deel van de stemmen en/of geluiden heb je in de afgelopen week als negatief ervaren?**

0: Nooit negatief; de hele inhoud is positief, nuttig of neutraal
1: Af en toe negatieve inhoud, maar dit is zelden het geval (<10%)
2: Een gedeelte van de inhoud is negatief
3: Ongeveer de helft van de inhoud is negatief
4: De meerderheid van de inhoud is negatief
5: Altijd, de hele inhoud is negatief

1.5. **Heb je in de afgelopen week wel eens last van de stemmen of geluiden gehad?**

0: Geen ongemak, het beïnvloedt me niet

1: Twijfel, misschien een beetje ongemakkelijk
2: Een beetje ongemakkelijk, de stemming of het gedrag kan worden beïnvloed
3: Aanzienlijk ongemak, het veroorzaakt soms een angstig, onrustig of depressief gevoel
4: Heel veel last, het veroorzaakt vaak een angstig, onveilig of depressief gevoel
5: Intense last, de stemmen of geluiden veroorzaken een ernstige depressie of angst

1.6. **Hebben de geluiden je dagelijks functioneren beïnvloed in de afgelopen week?**

0: Ze hebben geen effect op het uitvoeren van normale dagelijkse activiteiten
1: Ze beperken bij een aantal specifieke activiteiten, maar de meeste dingen kunnen worden uitgevoerd
2: Ze zijn beperkend bij diverse activiteiten
3: Ze weerhouden van de meeste activiteiten (bijvoorbeeld het huis niet verlaten)
4: Ze zijn zo verstorend dat het aanzet kan geven tot schelden, schreeuwen of vernieling
5: Ze veroorzaken verstoring van het dagelijks functioneren. Ziekenhuisopname of crisisopvang is mogelijk nodig

1.7. **Hoor je steeds dezelfde woorden, zinnen of geluiden?**

A: Geen herhalingen, steeds andere inhoud
B: Soms herhaling van de inhoud (woorden, zinnen of geluiden), maar veel afwisseling
C: Thema of inhoud wordt vaak herhaald, maar de woorden verschillen
D: Vaak herhaling van dezelfde woorden, zinnen of geluiden
E: Dezelfde woorden, zinnen of geluiden worden steeds herhaald, alsof de plaat blijft hangen

1.8. **Als je stemmen hoort, hoor je dan alleen losse woorden of een hele zin?**

(Meerdere antwoorden mogelijk)

A: Alleen non-verbale hallucinaties, geen stemmen
B: Eén los woord
C: Verschillende woorden
D: Korte zinnen
E: Een complete zin
F: Verhaal bestaand uit meerdere complete zinnen achter elkaar

1.9. **Waar komen de stemmen of geluiden vandaan?**

(Meerdere antwoorden mogelijk)

A: In het hoofd, geen specifieke plek in het hoofd
B: Vanuit een specifieke plek in het hoofd (bijvoorbeeld dichtbij het oor, in het voorhoofd, vanuit de mond)
C: Zowel in als buiten het hoofd
D: Buiten het hoofd, meestal van de linkerkant
E: Buiten het hoofd, meestal van de rechterkant
F: Buiten het hoofd, geen specifieke kant

1.10. **Op welk moment van de dag (of nacht) hoor je meestal de stemmen of geluiden**?

A: Geen specifiek patroon opgemerkt
B: Alleen ’s nachts
C: Voornamelijk in de avond
D: Rond het in slaap vallen en wakker worden
E: Voornamelijk overdag
F: Dag en nacht

1.11. **Mensen denken vaak verschillend over de oorzaak van de geluiden die ze horen. Sommige mensen zeggen dat ze mensen/wezens/geluiden horen die echt bestaan. Andere mensen zeggen dat de geluiden worden gecreëerd door de eigen hersenen. Wat denk jij dat maakt dat je de afgelopen week stemmen of geluiden hebt gehoord?**

0: Volledig van overtuigd dat de stemmen, geluiden of muziek **niet** echt zijn
1: Lichte twijfel of ze echt zijn, waarschijnlijk niet echt
2: Ze zijn waarschijnlijk echt, maar alternatieve verklaringen zijn ook mogelijk
3: Sterk overtuigd dat ze echt zijn, slechts minimale twijfel
4: Volledige overtuiging dat ze echt zijn

1.12. **Sommige mensen merken dat ze reageren op de stemmen of andere geluiden. Dat kan hardop of in stilte in gedachten. In de afgelopen week, hoe vaak heb je gemerkt dat je hebt gereageerd op de stemmen of geluiden?**

0: Nooit enige interactie gehad
1: Eén of enkele keren
2: Alleen wanneer de stemmen heftig zijn, normaal gesproken niet
3: Soms, maar niet altijd
4: Meerderheid van de tijd
5: (Bijna) altijd

1.13. **Als je de afgelopen week stemmen hebt gehoord, hebben de stemmen je dan wel eens opdrachten gegeven?**

□: Geen stemmen
0: Ik krijg nooit opdrachten van de stemmen
1: Ik krijg wel opdrachten, maar voer ze nooit uit
2: Ik voer soms onschuldige opdrachten uit (bijvoorbeeld tanden poetsen)
3: Ik voer altijd onschuldige opdrachten uit, maar geen gevaarlijke
4: Ik voer soms gevaarlijke opdrachten uit (bijvoorbeeld van de trap af springen)
5: Ik voer alle opdrachten altijd uit, ook de gevaarlijke

1.14. **Hoor je wel eens muziek of zingen terwijl de radio of televisie niet aanstaat of wanneer niemand anders het kan horen?** Zo ja, hoe vaak?

(Eén antwoord mogelijk)

A: Geen muzikale inhoud of zingen
B: Alleen een klein gedeelte van de geluiden is muzikaal, maar het grootste gedeelte is niet muzikaal
C: Ongeveer de helft van de geluiden is muzikaal
D: Het grootste gedeelte van de geluiden is muzikaal, maar stemmen of andere geluiden worden ook gehoord
E: Uitsluitend muzikale geluiden

1.15. **Sommige mensen horen wel eens de telefoon gaan, terwijl ze onder de douche staan. Als ze de douche uitzetten blijkt dit geluid er niet te zijn. Een ander voorbeeld is dat iemand een stem denkt te horen in het zoemen van de stofzuiger. Heb je zoiets wel eens ervaren?**
Zo ja, hoe vaak gebeurt dit?

0: Nooit tot minder dan eens per maand
1: Minimaal eens per maand
2: Minimaal eens per week
3: Minimaal eens per dag
4: Minimaal eens per uur
5: (Bijna) altijd

Appendix 2.1: additionele vragen indien item 2 positief is beantwoord.

2.1. **Hoe vaak zie je de beelden?**

0: Minder dan een keer per maand
1: Minimaal eens per maand
2: Minimaal eens per week
3: Minimaal eens per dag
4: Minimaal eens per uur
5: (Bijna) altijd

2.2. **a. Wat zie je precies? Kan je een voorbeeld geven?**

……………………………………………………………………………………………………

……

…………………………………………………………………………………………

b. Sinds wanneer zie je zulke beelden? Op welke leeftijd is dit begonnen?

……………………………………………………………………………………………………

……………………………………………………………………………………………………

c. Bij sommige mensen houdt dit verband met eerdere vervelende gebeurtenissen.
Is dit bij jou het geval? Ja/Nee

2.3. **Wanneer je de beelden in afgelopen week zag, hoe lang duurde dit toen?**

0: Heel kort; slechts een ogenblik
1: Een paar seconden
2: Een minuut of enkele minuten
3: 10 minuten tot een uur
4: Een uur tot een aantal uren
5: (Bijna) altijd

2.4. **Sommige mensen zien beelden met een negatieve inhoud. Welk deel van de beelden heb je in de afgelopen week als negatief ervaren?**

0: Nooit negatief; de hele inhoud is positief, nuttig of neutraal
1: Af en toe negatieve inhoud, maar dit is zelden het geval (<10%)
2: Een gedeelte van de inhoud is negatief
3: Ongeveer de helft van de inhoud is negatief
4: De meerderheid van de inhoud is negatief
5: Altijd, de hele inhoud is negatief

2.5. **Heb je in de afgelopen week wel eens last van de beelden gehad?**

0: Geen ongemak, het beïnvloedt me niet
1: Twijfel, misschien een beetje ongemakkelijk
2: Een beetje ongemakkelijk, de stemming of het gedrag kan worden beïnvloed

3: Aanzienlijk ongemak, het veroorzaakt soms een angstig, onrustig of depressief gevoel
4: Heel veel last, het veroorzaakt vaak een angstig, onveilig of depressief gevoel
5: Intense last, de beelden veroorzaken een ernstige depressie of angst

2.6. **Hebben de beelden je dagelijks functioneren beïnvloed in de afgelopen week?**

0: Ze hebben geen effect op het uitvoeren van normale dagelijkse activiteiten
1: Ze beperken bij een aantal specifieke activiteiten, maar de meeste dingen kunnen worden uitgevoerd
2: Ze beperken bij diverse activiteiten
3: Ze weerhouden van de meeste activiteiten (bijvoorbeeld het huis niet verlaten)
4: Ze zijn zo verstorend dat het aanzet kan geven tot schelden, schreeuwen of vernieling
5: Ze veroorzaken vermindering van het dagelijks functioneren. Ziekenhuisopname of crisisopvang is mogelijk nodig

2.7. **Zijn het telkens dezelfde mensen, patronen, dieren of dingen die je ziet?**

A: Geen herhalingen, steeds andere inhoud
B: Soms herhaling van beelden, maar veel afwisseling
C: Thema of inhoud wordt vaak herhaald, maar de exacte beelden verschillen
D: Vaak keren dezelfde beelden terug
E: Steeds herhaling van dezelfde beelden

2.8. **Wat voor soort beelden zie je precies?**

(Meerdere antwoorden mogelijk)

A: Simpele vormen zoals cirkels, lichtflitsen, stippen of lijnen
B: Patronen, zoals een schaakbord of diamantvormige objecten, rasters of blokken
C: (Vervormde) gezichten of schaduwen
D: Mensen en/of dieren
E: Levenloze dingen (bijvoorbeeld gebouwen, landschappen, voertuigen)

2.9. **Waar zie je de beelden meestal?**

(Meerdere antwoorden mogelijk)

A: Het kan overal zijn, geen overheersende locatie
B: Meestal aan de linkerkant
C: Meestal aan de rechterkant
D: Meestal recht voor me
E: Meestal in de ooghoek
F: In het gebied waar ik slecht zie

2.10. **Op welk moment van de dag (of nacht) zie je de beelden meestal**?

A: Geen specifiek tijdstip
B: Alleen ’s nachts
C: Voornamelijk in de avond
D: Rond het in slaap vallen en wakker worden
E: Voornamelijk overdag
F: Dag en nacht

2.11. **Mensen denken vaak verschillend over de oorzaak van de beelden die ze zien. Sommige mensen zeggen dat ze mensen/wezens/beelden zien die echt bestaan. Anderen mensen zeggen dat de beelden worden gecreëerd door de eigen hersenen. Wat denk jij dat maakt dat jij de afgelopen week de beelden hebt gezien?**

0: Volledig overtuigd dat de beelden **niet** echt zijn
1: Lichte twijfel of ze echt zijn, waarschijnlijk zijn ze niet echt
2: Ze zijn waarschijnlijk echt, maar alternatieve verklaringen zijn ook mogelijk.
3: Sterk overtuigd dat ze echt zijn, slechts minimale twijfel
4: Volledige overtuiging dat ze echt zijn

2.12. **Sommige mensen merken dat ze reageren op de beelden die ze zien, bijvoorbeeld door de beelden aan te raken of van ze weg te lopen. In de afgelopen week, hoe vaak heb jij gereageerd op de beelden die je ziet?**

0: Nooit gereageerd op de beelden
1: Eén of enkele keren
2: Alleen wanneer de beelden ernstig zijn, normaal gesproken niet
3: Soms, maar niet altijd
4: Groot gedeelte van de tijd
5: (Bijna) altijd

2.13. **In de afgelopen week, hebben de beelden je wel eens opdrachten gegeven? Of hebben de beelden geïmpliceerd dat je iets moet doen?**

0: Ik krijg nooit opdrachten van de beelden
1: Ik krijg wel opdrachten, maar voer ze nooit uit
2: Ik voer soms onschuldige opdrachten uit (bijvoorbeeld tanden poetsen)
3: Ik voer altijd onschuldige opdrachten uit, maar geen gevaarlijke
4: Ik voer soms gevaarlijke opdrachten uit (bijvoorbeeld van de trap af springen)
5: Ik voer alle opdrachten altijd uit, ook de gevaarlijke

2.14. **Heb je de afgelopen week wel eens een persoon of dier langs zien lopen, wat vervolgens verdween toen je er goed naar keek?** *Zo ja, hoe vaak gebeurt dit?*

(Eén antwoord mogelijk)

A: Nooit beelden gezien
B: Minder dan eens per maand
C: Minimaal eens per week
D: Minimaal eens per dag
E: Minimaal eens per uur

2.15. **Sommige mensen zien weleens een gezicht in de stam van een boom. Of een ander voorbeeld is dat mensen een hond zien in het patroon van het vloerkleed of het behang. Heb je zoiets wel eens ervaren?** Zo ja, *hoe vaak gebeurt dit?*

0: Nooit tot minder dan eens per maand
1: Minimaal eens per maand
2: Minimaal eens per week

3: Minimaal eens per dag
4: Minimaal eens per uur
5: (Bijna) altijd

Appendix 3.1: additionele vragen indien item 3 positief is beantwoord.

3.1. **Hoe vaak voel je dit?**

0: Minder dan een keer per maand
1: Minimaal eens per maand
2: Minimaal eens per week
3: Minimaal eens per dag
4: Minimaal eens per uur
5: (Bijna) altijd

3.2. **a. Wat voel je precies? Kan je mij een voorbeeld geven?**

……………………………………………………………………………………………………

……………………………………………………………………………………………………

……………………………………………………………………………………………………

b. Sinds wanneer voel je zulke dingen? Op welke leeftijd is dit begonnen?

……………………………………………………………………………………………………

……………………………………………………………………………………………………

c. Bij sommige mensen houdt dit verband met eerdere vervelende gebeurtenissen.
Is dit bij jou het geval? Ja/Nee

Appendix 4.1: additionele vragen indien item 4 positief is beantwoord.

4.1. **Hoe vaak ruik je dit?**

0: Minder dan een keer per maand
1: Minimaal eens per maand
2: Minimaal eens per week
3: Minimaal eens per dag
4: Minimaal eens per uur
5: (Bijna) altijd

4.2. **a. Wat ruik je precies? Kan je een voorbeeld geven?**

……………………………………………………………………………………………………

……………………………………………………………………………………………………

……………………………………………………………………………………………………

b. Sinds wanneer ruik je zulke geuren? Op welke leeftijd is dit begonnen?

……………………………………………………………………………………………………

……………………………………………………………………………………………………

c. Bij sommige mensen houdt dit verband met eerdere vervelende gebeurtenissen.
Is dit bij jou het geval? Ja/Nee
